# Relative pose estimation from panoramic images using a hybrid neural network architecture

**DOI:** 10.1038/s41598-024-75124-7

**Published:** 2024-10-24

**Authors:** Lars Offermann

**Affiliations:** Faculty of Technology, Bielefeld University, 33615 Bielefeld, Germany

**Keywords:** Imaging and sensing, Computer science

## Abstract

Camera-based relative pose estimation (RPE) localizes a mobile robot given a view at the current position and an image at a reference location. Matching the landmarks between views is critical to localization quality. Common challenges are appearance changes, for example due to differing illumination. Indirect RPE methods extract high-level features that provide invariance against appearance changes but neglect the remaining image data. This can lead to poor pose estimates in scenes with little detail. Direct RPE methods mitigate this issue by operating on the pixel level with only moderate preprocessing, but invariances have to be achieved by different means. We propose to attain illumination invariance for the direct RPE algorithm MinWarping by integrating it with a convolutional neural network for image preprocessing, creating a hybrid architecture. We optimize network parameters using a metric on RPE quality, backpropagating through MinWarping and the network. We focus on planar movement, panoramic images, and indoor scenes with varying illumination conditions; a novel dataset for this setup is recorded and used for analysis. Our method compares favourably against the previous best preprocessing method for MinWarping, edge filtering, and against a modern deep-learning-based indirect RPE pipeline. Analysis of the trained hybrid architecture indicates that neglecting landmarks in a direct RPE framework can improve estimation quality in scenes with occlusion and few details.

## Introduction

Appearance changes in the scene challenge the accuracy and robustness of relative pose estimation (RPE) from camera images. RPE is used to localize mobile robots and serves as a foundation for higher-level tasks like navigation and map-building. Therefore, improving RPE benefits agents on a fundamental level.

In general, the time between capturing the snapshot and the current view may be arbitrarily long, for example when navigating with a map of previously visited places. When the illumination in the scene varies between capture positions, pixel values for a view of the same environment change drastically.

While indirect approaches to RPE detect and describe high-level image features that provide tolerance against appearance changes, direct approaches use pixel values, allowing to use the whole data in the image (see “Related work”). This is beneficial in scenes with few details but well visible contours^1^, but robustness against global illumination must be achieved by other means.

This is a key challenge for direct RPE methods that allow for arbitrary durations between image captures. We focus on one such method, MinWarping^2^, which is designed for panoramic images and based on a planar motion assumption.

Previous attempts to achieve illumination tolerance for MinWarping use edge filtering to reduce lighting sensitive information in the input images^3^, but the RPE quality is still noticeably worse than in situations with constant illumination.

Deep neural networks can be a possible solution as they excel at computer vision tasks like classification and image generation, but large architectures require large datasets for training. Reference data for estimating the pose of mobile robots is costly, because the gathering process depends on specialized hardware and often also human supervision. Instead of modelling RPE using a neural network, we develop a hybrid architecture by extending MinWarping with a convolutional neural network (CNN) as an image preprocessing step. We present a loss function that enables training with backpropagation through the RPE algorithm by smoothly approximating the discretized pose estimates of MinWarping. To the best of our knowledge, a hybrid architecture that integrates the RPE algorithm into the training process is a novel approach to robot localization.

Besides the reduced demand of training data, limiting the domain of the neural network to image preprocessing and using a manually designed algorithm for RPE allows for easier interpretability compared to a fully machine-learning based approach. We use this for an analysis of the network output in conjunction with the RPE algorithm.

Our description of MinWarping and the following evaluation are aimed at local visual homing in indoor environments as an application of RPE to robot navigation. Local visual homing uses two images called a snapshot and a current view to estimate the relative pose, then steer the robot in the direction of the capture location of the snapshot. To train the network and evaluate the RPE quality of the selected methods, we use a novel panoramic image dataset gathered in home and office environments.

**Figure 1:**
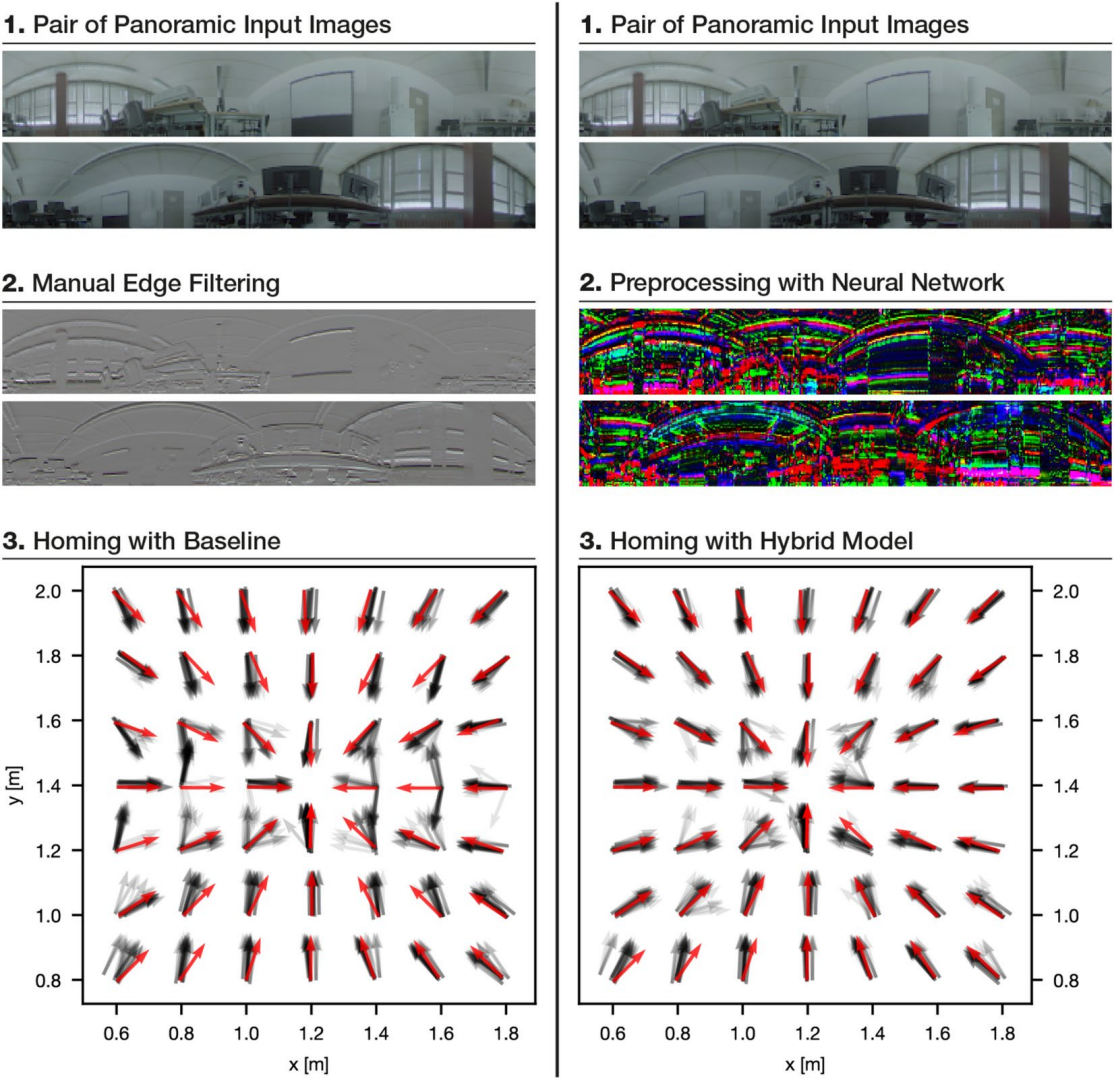
Comparison of MinWarping with neural network image preprocessing (proposed method, right) against MinWarping with edge filtering (left). Example image pairs show a home position (“snapshot”) and a changed position after robot movement (“current view”). All images are represented in RGB color space. Homing examples show a $7\times 7$ section of a grid image dataset with the snapshot position marked with a red dot and reference homing vectors as red arrows. Gray arrows are overlayed to show the homing results for each current view with respect to the snapshot, considering all illumination variants present in the dataset and positions from ground truth data. All data is from setting “Computer Lab”; see “Dataset”.

We compare the proposed hybrid architecture against MinWarping with edge filtered input images. To this end, we find high-dynamic range (HDR) color images to be the favourable input image type for MinWarping. Examples for the type of image data and homing results are shown in Figure 1. As an additional point of reference, we compare the pose estimation quality for both direct methods with that of a recent deep-learning-based pipeline for indirect RPE as presented by Lindenberger et al.^4^.

The remainder of this paper starts with an overview of related work in the “Related work” section. The “Dataset” section contains a description of the panoramic image dataset, the capturing process and the steps used to preprocess the data for training and evaluation. Then, we introduce MinWarping as our choice of RPE method in “Visual homing and MinWarping” and outline the hybrid image preprocessing architecture in “Network-based image preprocessing”. We continue with a description of the performance metrics Average Angular Error (AAE) and Return Ratio (RR) in “Metrics”. Following that, we present qualitative and quantitative results, including a comparison of execution times in “Results”. Results are discussed in the “Discussion” section. We close with a conclusion in the “Conclusion” section.

## Related work

We consider relative pose estimation (RPE) from monocular images in the context of visual robot navigation systems, which can be categorized as map-less, map-based, and map-building^5^. Besides local visual homing, visual odometry^6,7^ is an example of a map-less application. Visual odometry algorithms iteratively estimate the trajectory of a robot from high frequency image sequences, tracking features from frame to frame. The pose estimate is the result of an optimization using gradient descent. In constrast, local visual homing uses RPE from image pairs to navigate to a snapshot position and can work with arbitrarily long time differences between images.

We classify solutions to RPE by their reliance on geometric principles.

### Geometry-based RPE methods

Geometry-based methods exploit the relation between a point in the scene and its 2D projections to at least two camera images; this is referred to as epipolar geometry^8^. Both direct and indirect algorithms rely on these geometric principles.

#### Indirect RPE methods

Indirect RPE is realized as a pipeline that starts with the detection, description, and matching of points in the input images. Feature extraction can account for deficiencies in the image capturing process, for example lens distortions and exposure changes, and provide invariances to appearance changes like scale, rotation, and illumination. Feature detectors focus on salient image details and neglect the remaining pixels, which is challenging in environments with few available features^1^. An additional problem are mismatched feature descriptors due to motion blur, appearance similarity, or occlusion^9^. As a countermeasure, RANSAC^10^ is used to eliminate outliers. Hereby, the relative camera pose is estimated from a set of point pairs by fitting a geometric model under epipolar constraints by minimization of a reprojection error^8^.

Examples for manually designed feature detectors and descriptors are SIFT^11^, SURF^12^, and ORB^13^. Recently developed alternatives employ machine learning for feature detection^14^, detection and description^15–19^, matching^4,20,21^, or all of the above^22^. For an overview of additional methods on detection and description, see the work of Luo et al.^19^. We select an indirect RPE pipeline based on SuperPoint^16^, LightGlue^4^ and RANSAC^10^ as a comparison to both MinWarping methods studied here. According to Lindenberger et. al.^4^, this pipeline achieves better results than a similar one using SIFT and is competitive to other indirect machine-learning-based RPE methods.

#### Direct RPE methods

Direct RPE methods pose an alternative to indirect RPE that use little or no feature detection. Instead of minimizing a reprojection error, they employ a parameterized image warping function and optimize a pixel-wise similarity score. The image warping function relies on depth estimates or additional assumptions regarding the distance of the camera to the landmarks. Advantages with respect to indirect methods include the computationally inexpensive image preprocessing step and the possibility to operate in scenes with few details but well-visible contours^1^. However, the lean image preprocessing cannot not provide the same type of invariances achieved by feature extractors. Depending on the amount of feature detection used, direct methods can be separated into sparse and holistic methods: Sparse direct methods detect salient points by inexpensive means. An example is the visual odometry method DSO^23^, which selects trackable points using thresholds on an edge filtered image. Holistic approaches use all available image pixels without selecting salient points, e. g. DTAM^24^ (visual odometry), or MinWarping^2^ (visual homing in the plane).

### Machine learning for RPE

Approaches to RPE that do not actively rely on epipolar geometry use machine learning and either estimate the camera pose from sequences or image pairs.

#### Machine learning architectures for RPE using image pairs

Existing image-pair-based models use supervised learning to train siamese network architectures with two CNN-based feature extractors and a densely connected regression network^25–27^. Feature extraction is handled by pre-trained architectures, for example for classification (GoogLeNet/Inception V1^28^ in the work by En et al.^26^ and VGG^29^ as explored by Kamranian et al.^27^) or scene recognition (Hybrid-CNN^30^ used by Melekhov et al.^25^). Transfer learning enables training with restricted data availability. We evaluated the method described by Melekhov et al.^25^ as an example of a siamese network architecture, but found it unsuitable for the image data used in this work. For details, see Section S1 in the Supplementary Information.

#### Machine learning architectures for RPE using image sequences

Machine learning models that continuously estimate camera poses from image sequences are either trained supervised using pose data as ground truth^9,31,32^ or unsupervised with video prediction as a surrogate task^33–35^. Notably, the works of Zhan et al.^36^ and Yang et al.^37^ show that combining traditional geometry-based methods for RPE with deep learning can lead to hybrid models that exceed the pose estimation quality of purely geometry-based and purely deep-learning based methods (see the work of Yang et al.^37^ for an overview and comparison). Contrary to our approach, these methods focus on image sequences with short temporal distances instead of image pairs and train machine-learning-based parts (depth, pose, uncertainty estimation) separately from the manually designed algorithm.

## Dataset

As the basis for our study, we collected a dataset of real-world images with a focus on illumination changes. Every data point consists of a multi-exposure HDR color image, the associated exposure series of panoramic images, and the position of the camera in the plane. All images in the database have the same orientation and get rotated in azimuthal direction before being passed to the RPE methods.

**Figure 2:**
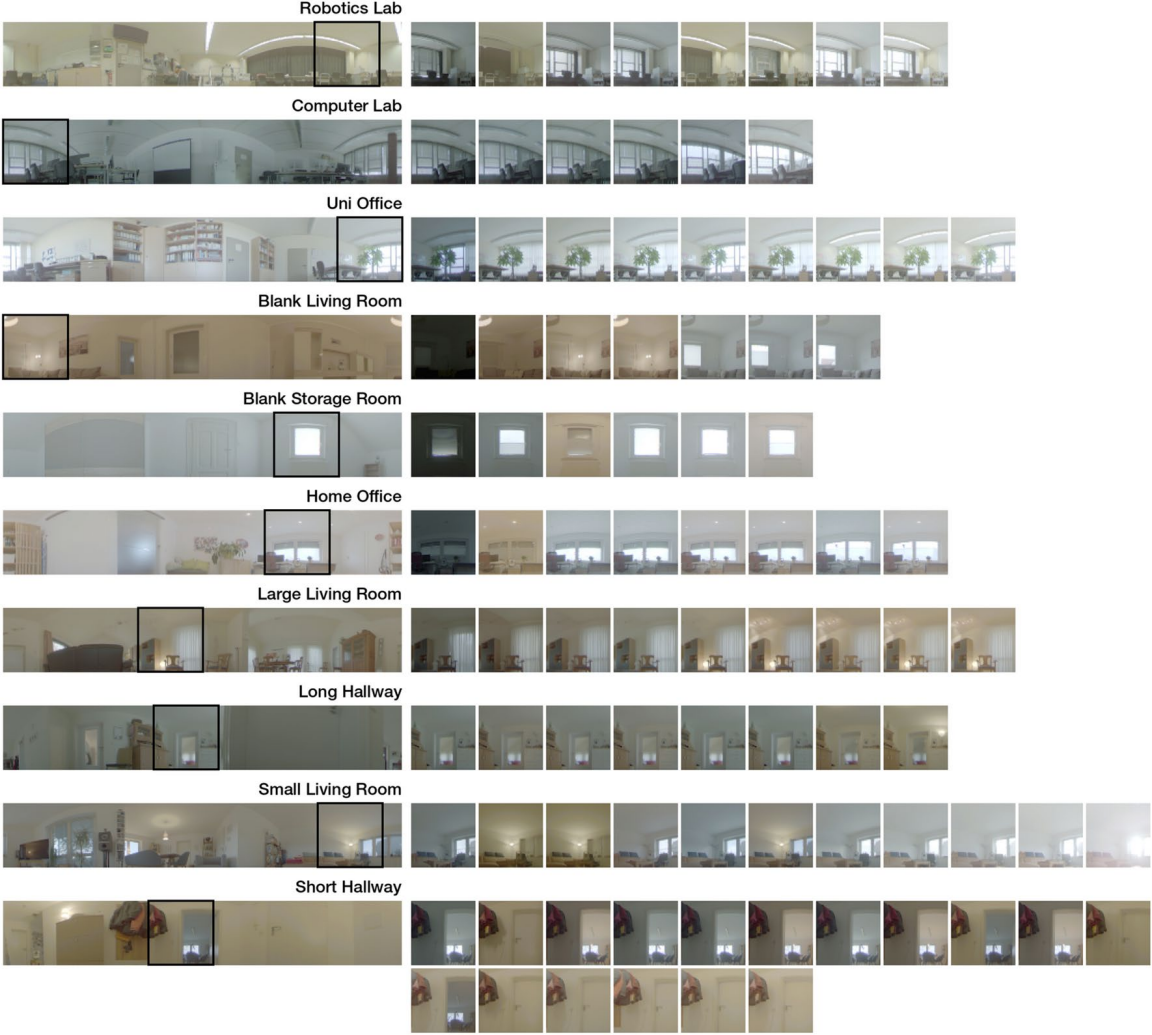
Overview of illumination variants for each setting. All images are located at grid index (3, 3). On the left side, we show a reference image as it gets used for network input. A distinctive detail that well reflects illumination changes in the room is marked with a black box. On the right side, the detail is shown for all recorded illumination variants, sorted by average brightness. All images are mapped from HDR using the logarithmic tonemapping described in the “Preprocessing” section.

We recorded data points on a rectangular grid with a target spacing of 20 cm in each direction. For every grid point, we collected a series of data points with varying illumination. A collection of data points in the same scene is called a *setting* and an instance of illumination within a setting is called a *variant*. Each setting covers a rectangular, obstacle-free section of a single indoor space. Variants are selected to capture the typical illumination in the environment, for example alterations of artificial illumination, natural light, and differing daytime/weather. Aside from changes in illumination, we kept the enviroments as static as possible. We recorded datasets in 10 typical environments for indoor robots at home and in offices/laboratories Bielefeld University. Overviews are provided in Table 1 and Figure 2. The total number of recorded images is 7222.

In the following, we describe how the datasets were collected, processed, and partitioned into training, validation and test splits.

**Table 1:** Overview of dataset with descriptions of settings. “Number of variants” is abbreviated to “#Var”.

Setting	Grid size	#Var.	Description	Light sources
Robotics Lab	$12 \times 12$	8	Mobile robotics lab in the main building of Bielefeld University with changing weather and daytimes. The object quantity is high and their distribution is even.	Windows with opaque curtains, shutters, ceiling lamps.
Computer Lab	$12 \times 11$	6	Computer lab with different weather conditions. The object quantity is medium. There are fewer objects at the wall with the projection screen. Otherwise, the object distribution is even.	Shutters, beamer, portable point light.
Uni Office	$13 \times 8$	9	Office in the main building of Bielefeld University with high object quantity and even distribution.	Windows with translucent curtains, open door to an adjacent room, ceiling lamps.
Blank Living Room	$7 \times 9$	7	Sparsely decorated living room. The number of objects is low, but except close to doorways, they are evenly distributed.	Windows with shutters and blinds, hanging, floor lamps.
Blank Storage Room	$10 \times 9$	6	Sparsely furnished room containing only a bookshelf and a wardrobe. The number of objects is low, but they are evenly distributed.	Single window with blinds, ceiling lamps.
Home Office	$7 \times 10$	8	Two-desk office at home. High object quantity and even distribution with the exception of areas close to doorways.	A single large window with blinds and shutters, glass door to adjacent room, ceiling lamps.
Large Living Room	$7 \times 10$	9	Living/dining area with a high number of objects and even distribution with the exception of the back of the sofa.	Windows with translucent curtains, ceiling, hanging, floor lamps, table lamp.
Long Hallway	$19 \times 4$	8	Long hallway with a narrow and a wide section. The number of objects is medium, and the density is only high at the two ends of the elongated room.	Outside-facing windows, windowed doors to adjacent rooms, ceiling, wall and table lamps.
Small Living Room	$11 \times 8$	11	Living/dining area. Variants include changes of daylight and mixed weather. The object quantity is high and their distribution is even.	Windows and outside-facing glass doors with blinds, floor, table and hanging lamps; indirect illumination from adjacent rooms.
Short Hallway	$5 \times 7$	17	Small, rectangular hallway with no windows; variants are split into two classes: illumination from adjacent rooms and illumination from within. The object quantity is low and the object density is concentrated on one side of the room with a wardrobe and a coat rack.	Four adjacent rooms through opened doors, ceiling, table and floor lamps.

### Dataset collection

**Figure 3:**
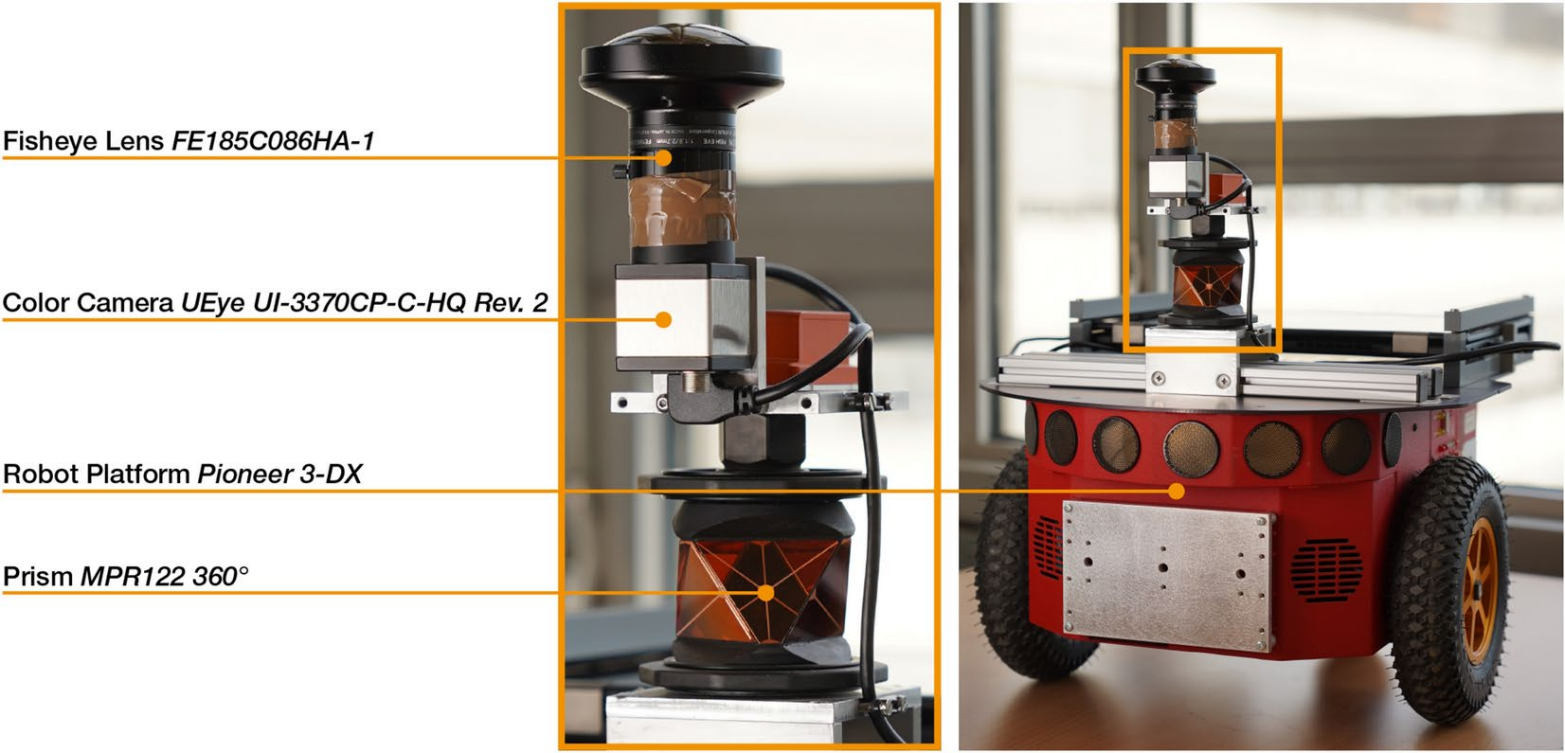
Depiction of the Omron Adept Pioneer 3-DX robot used to capture the image dataset. The left image shows the sensors in detail. The manufacturer logo on the camera body was removed using image processing.

We capture image datasets by means of an Omron Adept Pioneer 3-DX robot, a platform actuated by two independently controlled wheels and stabilized by a caster wheel as depicted in Figure 3.

The position of the robot is measured in terms of a global coordinate system using a Leica TS16 3” R500 robotic total station that tracks a Leica MPR122 360° prism mounted at the center of rotation between the two main wheels. If the total station is moved between variants, we re-measure predetermined landmarks in the scene to keep the coordinate system persistent. All measurements are taken indoors, with a distance of less than 10 m between the total station and the prism. Therefore, the positional error is mainly determined by the precision of the distance measurement of the instrument, which is specified with a standard deviation of 2 mm (fast measurements on prism).

The powered wheels of the robot are equipped with a wheel encoder, which we use to estimate the orientation of the robot in local coordinates. Because this estimate drifts, we correct the orientation on straight path segments by continously fitting lines through the last seven position measurements from the total station with a distance of at least 1 mm, then using the orientation of the line as the new estimate.

We record grid points of a variant by planning meandering trajectories that coincide with the desired grid points and use a trajectory controller to autonomously drive the robot. We record a data point at the time the planned path reaches the associated grid point, relying on the controller to minimize the positional error between desired and actual trajectory. For the recorded databases, the planar distance of the trajectory controlled robot to the desired grid points is 1.02 cm on average with a standard deviation of 0.49 cm and a maximum of 7.70 cm.

Images are captured using an IDS UEye UI-3370CP-C-HQ Rev. 2 color camera that is mounted at the highest point of the robot, facing upward. We use a Fujifilm FE185C086HA-1 circular fisheye lens for C-Mount with a 185° opening angle, set to a fixed aperture of F1.8. The the camera setup is manually leveled with respect to the floor using a circular level. During recording, we use two different camera bodies of the same type, the second one replacing a defective first unit. We determine an intrinsic calibration for each unit using the method of Urban et al.^38^. The sensor gain of both cameras is set to the fixed values 130, 100, 166 (considering 8-bit images) for red, green, and blue channels.

With the goal of computing high dynamic range (HDR) images, we capture exposure brackets for every data point with 10 exposure steps starting from $\sim$500 ms and successively halving the exposure time. Using the dual exposure mode of the camera, two differently exposed 8-bit images are taken at the same time with a resolution of $2048 \times 1024$ pixels per image. In the following, we refer to a single image of the exposure series as low dynamic range (LDR) image.

We compute camera response functions for HDR calculation using two variants of the setting Uni Office at grid position (0, 0) with the method described by Robertson et al.^39^. We set the parameter $W$ for the corresponding weighting function to 20 based on the subjective quality of the resulting HDR images.

### Preprocessing

We process raw positional and image data in two phases: before any training or evaluation occurs (offline), and continously during usage (at runtime).

#### Offline processing

During the offline phase, we first convert capturing positions to a right-handed global coordinate system. Preprocessing of every data point begins by calculating integer grid indices from continuous positions in global coordinates. Then, every dual exposure image of the series is split into two single exposure images, resulting in a total of 10 8-bit images with 1024 vertical and 2048 horizontal pixels. All images in the exposure series are then converted to a single-precision floating point format in the range $[0,1]$, low-pass filtered using a third-order Butterworth filter with zero-shift and a relative width of 0.2 to reduce aliasing artifacts during resampling, and clipped channel-wise to the range $[0,1]$. Next, the fisheye images are remapped to 360° cylindrical panoramic views in azimuth-elevation format using nearest-neighbor interpolation and the intrinsic calibration; for examples, see Figure 2. This includes rotation of images such that the local coordinate system for each data point is aligned with the global coordinate system. The remapping creates a reversed angle direction, therefore steps in positive pixel direction along the horizon correspond to negative angles. We selected an image width of 384 columns; the number of rows is calculated automatically such that angle per pixel is identical in both directions, resulting in an image height of 99 px. The resolution is $\sim$0.0163 rad/px. Completing the preprocessing, every LDR image is converted back to an 8-bit format. For HDR, we use the algorithm described by Robertson et al.^39^ to estimate an irradiance map from the processed LDR images using the precomputed camera response functions. The resulting HDR image is represented using a single-precision floating point format.

For pixels that were overexposed in every LDR image, resulting HDR values are exclusively determined by sensor noise and therefore cause artifacts. To mitigate this issue, we compute dataset-global maximum and minimum values for all HDR color channels. Then, we determine under- and overexposed pixels by converting associated LDR images to a single gray channel, calculating a pixel-wise mean over all exposures, then setting all pixels in the HDR image that had a mean in the exposure series above 247 (considering 8-bit pixels) to the predetermined maximum value. This method is adapted from the work of Differt^40^.

All 10 LDR images and the combined HDR image are losslessly saved alongside meta data for the real-world 2D position, integer grid position, and exposure times in an HDF5 format.

#### Processing at runtime

At runtime, the preprocessed grid data points are arranged into pairs of snapshot and current view and organized into disjoint collections to enable training, validation (for assessment of different hyperparameters), and testing (final evaluation).

We choose the whole setting Computer Lab to test our method on an unseen scenario, because the homing quality of MinWarping with edge filtering for this setting is average in comparison to the other settings (see “Average angular error (AAE) by setting”), suggesting that the change of illumination is neither trivial nor extreme.

For training and validation datasets, we first generate a set that contains all possible variant combinations for each setting as triplets of the form *(Setting, Variant for Snapshot, Variant for Current View)*. This set is then split into two subsets, with 90% of triplets used for training and the other 10% used for validation. Each of the two subsets of triplets gets processed independently: First, we find all image pairs setting-wise, excluding pairs at the same grid position. Next, the sets of image pairs for each setting are shuffled. For training, we then build the final set of samples by selecting 36000 pairs per setting, oversampling by cyclic repetition if less pairs are available. For validation, we collect all available image pairs.

When evaluating the homing quality on the whole dataset as in “Selecting the optimal image preprocessing using MinWarping with edge filtering and Homing quality comparison”, we select all image pairs from all available variants, excluding pairs at the same image position.

We shuffle training, validation, and test datasets once. A fixed seed is used to ensure comparability between experiments.

For training or evaluation, we load either HDR or LDR images depending on the experiment. The upper 35° of the vertical field of view is then cut off to discard areas with large amount of distortion. This was determined using a parameter search (see Section S3 in the Supplementary Information). The resulting number of rows is 62 and the image horizon is located at row $\sim$59.32, measured from the top of the image. We convert HDR to log-space and normalize to the range $[0,1]$ to achieve a parameter-free tonemapping.

In “Selecting the optimal image preprocessing using MinWarping with edge filtering”, we test different image preprocessing techniques for MinWarping with edge filtering. Hereby, we compute histogram equalization for LDR images on the value-channel of the corresponding HSV color image. Emulating an exposure time controller, we select the image from the exposure series whose average gray value over the whole pixel range is closest to 40. If grayscale images are needed, we convert the color images using the default weights provided by Tensorflow^44^: $0.2989, 0.5870, 0.1140$ for red, green, and blue channels.

Finally, images are shifted along the horizontal axis by randomly selected integer values $r_s, r_c$, simulating a rotation of the robot platform at both the snapshot and current location. Consequently, ground truth values for an image pair are computed as

1 $$\begin{aligned} \psi = \frac{2\pi }{w} (r_s - r_c),\quad \alpha = \frac{2\pi }{w}r_s-\text {atan2}(y_c - y_s, x_c - x_s). \end{aligned}$$

We restrict $\alpha$ and $\psi$ to the range $[0, 2\pi ]$.

## Visual homing and MinWarping

In the following, we introduce the geometric principles used in 2D homing and their application in the MinWarping algorithm.

### Overview of geometric constraints for 2D homing

We focus on homing in the plane using pairs of the 360° panoramic images introduced in the “Dataset” section: a snapshot image $\varvec{S}$ and a current view $\varvec{C}$.

**Figure 4:**
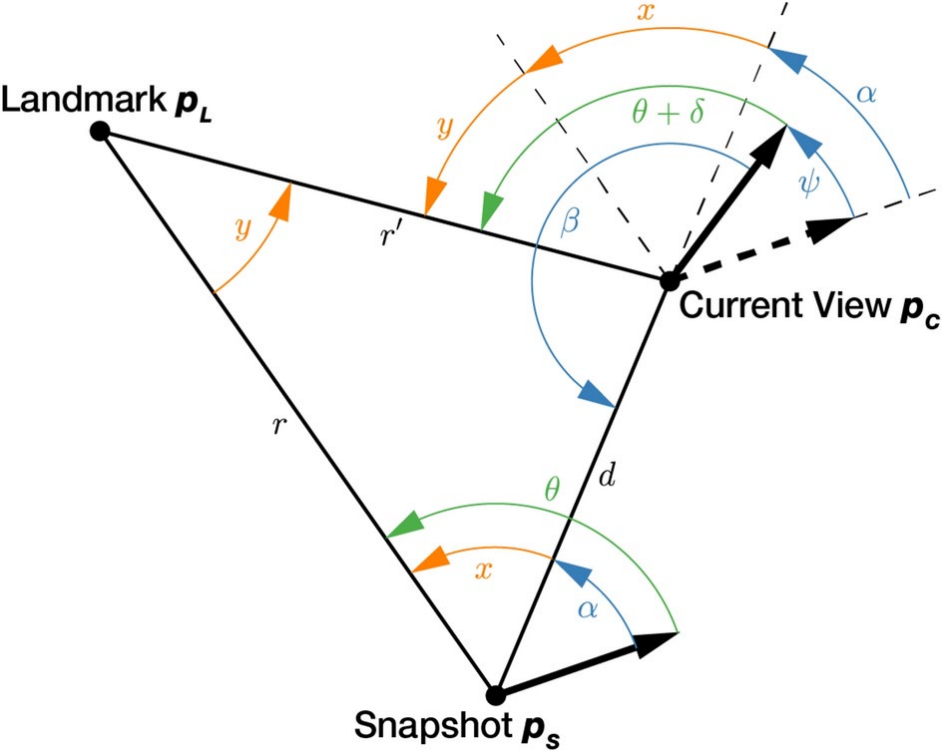
Geometry of 2D homing. Sets of angles are color coded as follows: green: landmark bearing angle from image origin, orange: landmark bearing angle from focus of expansion (FOE), blue: movement parameters to search for. Figure adapted from Möller et al.^2^.

To estimate the relative pose of the robot in the form of two movement parameters, we exploit the triangular relation between the current position of the robot $\varvec{p_C}$, its previous position $\varvec{p_S}$, and the position of a landmark $\varvec{p_L}$ as shown in Figure 4. Positions refer to 2D coordinates in reference to a global coordinate system. For 2D homing, we consider every column of a cylindrical panoramic image as a landmark.

Assuming static surroundings, a translation of the robot in the plane creates an optical flow between $\varvec{S}$ and $\varvec{C}$ with distinct properties: there is a focus of expansion (FOE) in the movement direction and a focus of contraction (FOC) at the opposite side of the circular image. Landmarks generally move from FOE to FOC. When the viewing distance to the landmark changes from $r$ to $r'$, the landmark also appears differently: Considering the view from the snapshot position $\varvec{p_S}$, the landmark scale decreases if the ratio $\frac{r'}{r}$ is less than 1 and increases for $\frac{r'}{r}$ larger than 1.

We define the forward direction of the robot as an azimuth of 0 in image coordinates for both $\varvec{S}$ and $\varvec{C}$. Then, we observe the landmark $\varvec{L}$ from the snapshot position $\varvec{p_S}$ at an angle $\theta$, which changes to $\theta +\delta$ at the current view position $\varvec{p_C}$. Angles relative to the origin in image coordinates are marked green in Figure 4.

Based on the images $\varvec{S}$ and $\varvec{C}$, the homing process estimates the two angular movement parameters $\alpha$ and $\psi$. $\alpha$ is an estimate of the angle between the forward direction of the robot at the snapshot position $\varvec{p_S}$ and the direction of translation from $\varvec{p_S}$ to $\varvec{p_C}$. In image coordinates, $\alpha$ corresponds to the azimuth angle between the origin and the FOE. The angle $\psi$ describes an estimate of the change of azimuthal orientation at $\varvec{p_C}$ with respect to the orientation at $\varvec{p_S}$, representing an “image compass”. From these values, we compute the homing angle as $\beta = \pi + \alpha -\psi$, describing the return direction from $\varvec{p_S}$ to $\varvec{p_C}$. The movement parameters $\alpha$ and $\psi$ are marked blue in Figure 4.

Geometric constraints for the optical flow of landmarks relate to the FOE. We observe the landmark $\varvec{L}$ in $\varvec{S}$ at an angle $x = \theta - \alpha$ from the FOE and the difference in bearing angle to the same landmark in $\varvec{C}$ as $y = \delta + \psi$. We disregard landmarks directly in the FOE or FOC as they provide no benefit to motion estimation: $x \in (-\pi ,\pi )\setminus \{0\}$. The angle range of $y$ is constrained by $x$: Since landmarks move towards FOC from the FOE, the positive angle range $y$ must be at least as large as $x$ and can be at most as large as $\pi$. The same is true for the negative angle range, yielding

2 $$\begin{aligned} y \in \left\{ \begin{array}{ll} {[}0,\pi -x] &  x \in (0, \pi )\\ {[}-\pi -x,0] &  x \in (-\pi , 0) \end{array}\right. . \end{aligned}$$

### MinWarping

Instead of explicitly warping images using a function derived from these geometric constraints as described by Franz et al.^41^, the MinWarping algorithm^2^ first computes all column-wise distances between $\varvec{S}$ and $\varvec{C}$, then searches for the parameters $\alpha$ and $\psi$ that best describe the observed landmark movement.

#### Phase 1: Column-wise distances

We represent images for snapshot and current view as three-dimensional arrays $\varvec{S, C} \in [0,1]^{h\times w\times c} \subset \mathbb {R}^{h\times w\times c}$. Hereby, $h, w,$ and $c$ describe the height (number of rows), width (number of columns), and color channels, respectively. The $i$-th image column is a slice along the second dimension of the image array and is denoted $\varvec{S}_{1:h,i,1:c}$. The slice is a two-dimensional array of size $h \times c$. We compute the distance matrix $\varvec{P}'$ by applying a distance function $d(\varvec{A}, \varvec{B})$ to all combinations of image columns:

3 $$\begin{aligned} \varvec{P}'_{i(\delta ),i(\theta )} = d({\varvec{C}}_{1: h,i(\theta +\delta ),1:c},{\varvec{S}}_{1:h,i(\theta ),1:c}) \end{aligned}$$

with $\theta , \delta \in [0,2\pi ]$ and $i: [0,2\pi ] \rightarrow [1, ..., w]$ as a function that discretizes angles into column indices. Since the observation angle $\theta + \delta$ for the landmark in $\varvec{C}$ is relative to the observation angle $\theta$ of the same landmark in $\varvec{S}$, the indexing into $\varvec{C}$ is shifted, giving the resulting distance image a skewed appearance. Following Möller^42^, we use the normalized sum of absolute distances (NSAD) as a distance function

4 $$\begin{aligned} d(\varvec{A}, \varvec{B}) = \sum _c \frac{\sum _r |\varvec{A}_{r,c}-\varvec{B}_{r,c}|}{\sum _r (|\varvec{A}_{r,c}|+|\varvec{B}_{r,c}|)}. \end{aligned}$$

with $r$ indexing into the image row and $c$ selecting a channel. In our implementation, we add $10^{-7}$ to the numerator to prevent undefined values.

**Figure 5:**
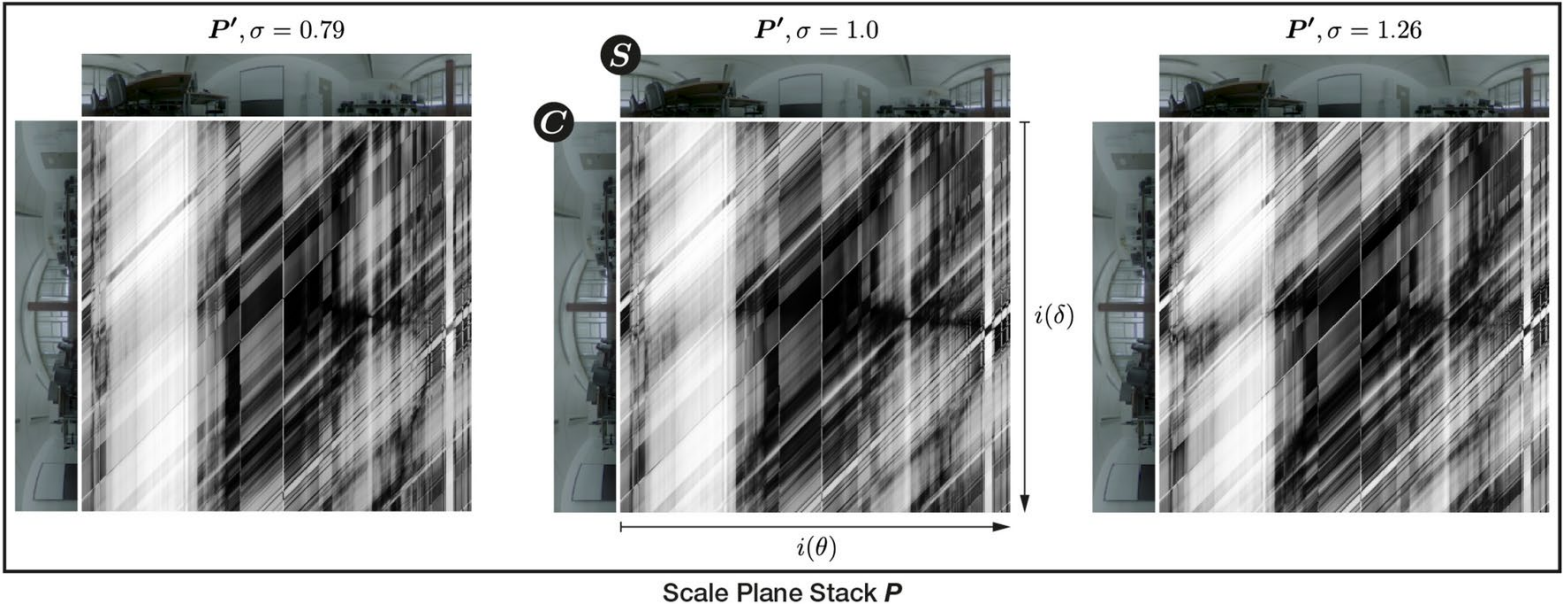
Example of the computation of the scale plane stack $\varvec{P}$ with three scales $\sigma \in \{0.79, 1.0, 1.26\}.$ For this demo, we compute distance images directly from RGB data. We use images from the dataset introduced in “Dataset” (setting “Computer Lab”) with constant illumination between snapshot $\varvec{S}$ and current view $\varvec{C}$. The image selection simulates a movement of the robot along the y-axis of the world coordinate sytem by $\sim$0.6 m, followed by a rotation of 180°. The values of the scale plane stack are treated with histogram equalization to improve visibility.

In practice, distances to landmarks $r$ and $r'$ vary. To support this, we compute additional image distance matrices by upscaling either $\varvec{S}$ or $\varvec{C}$ around the image horizon using a discrete set of scaling factors $\sigma = \frac{r'}{r}$. For $\sigma < 1$, $\varvec{S}$ is upscaled by $1/\sigma$, whereas for $\sigma > 1$, $\varvec{C}$ is upscaled by $\sigma$. For $\sigma = 1$, both images are used without scaling. We upsample using nearest-neighbor interpolation. This first phase results in a stack of distance images called a *scale plane stack* $\varvec{P} \in \mathbb {R}^{n_\sigma \times w \times w}$ with $n_\sigma$ as the number of scales and $w$ as the input image width. Therefore, $\varvec{P}$ is a collection of $n_\sigma$ distances matrices $\varvec{P'}$ with rescaled snapshot or current view images. An example is shown in Figure 5. Throughout this work, we use the inversion-symmetric scaling factors $\sigma \in \{0.5, 0.63, 0.79, 1.0, 1.26, 1.59, 2.0\}$ (rounded to two decimal places).

#### Phase 2: Search for optimal movement parameters

In phase 2, we optimize the movement parameters $\alpha , \psi$ using a grid search with $n_\alpha$ steps for $\alpha$ and $n_\psi$ steps for $\psi$. For each discrete instance of $\alpha , \psi$, we translate from the scale plane stack indices $\theta , \delta$ to the bearing angles $x, y$ relative to the FOE by applying $\alpha , \psi$ as offsets. Since $r$ and $r'$ may differ for every landmark, the corresponding scaling $\sigma$ for every combination of $x$ and $y$ can be determined using the law of sines: $\sigma =\frac{r'}{r}=\frac{\sin (x)}{\sin (x+y)}$. This leads to a set of indices $Q$ into $\varvec{P}$:

5 $$\begin{aligned} Q= &   \Bigg \{(j(\sigma ), i(y), i(x)) \Bigg | x \in (-\pi ,\pi )\setminus \{0\}, y \in \Bigg \{\begin{array}{ll} {[}0,\pi -x] &  x \in (0, \pi )\\ {[}-\pi -x,0] &  x \in (-\pi , 0) \end{array} \Bigg \}, \end{aligned}$$

6 $$\begin{aligned} x= &   \theta - \alpha ,\quad y = \delta + \psi ,\quad \sigma =\frac{r'}{r}=\frac{\sin (x)}{\sin (x+y)} \end{aligned}$$

The function $j: \mathbb {R}_{>0} \rightarrow [1,...,n_\sigma ]$ converts from a scaling factor to the associated index in the scale plane stack, with $\mathbb {R}_{>0}$ denoting the set of real numbers larger than zero.

To get the total score for an instance of $\alpha , \psi$, we match columns; for every column with an angle $x$, we find the column $y$ with the lowest distance and add the results. We achieve this by first splitting $Q$ into subsets with identical values of $x$, then finding the minimum in the values from $\varvec{P}$ that belong to the indices in the subset:

7 $$\begin{aligned} \varvec{D}_{i_\psi (\psi ),i_\alpha (\alpha )} = \sum _x \min _{y,\sigma } \{\varvec{P}_{(j(\sigma ), i(y), i(x))} | \big (j(\sigma ), i(y), i(x)\big ) \in \text {Q}\} \end{aligned}$$

with $i_\alpha : [0,2\pi ] \rightarrow [1, ..., n_\alpha ]$, $i_\psi : [0,2\pi ] \rightarrow [1, ..., n_\psi ]$ as functions that translate from angles to indices into $\varvec{D}$.

The result of this grid search is a matrix $\varvec{D}$ of size $n_\alpha \times n_\psi$. Selecting the minimum in this matrix yields the indices corresponding to the optimal movement parameters $\alpha , \psi$.

#### Extensions to the MinWarping algorithm

We use two extensions to the base MinWarping algorithm: double search^43^ and edge filtering.

For double search, we swap the snapshot and current view by creating a second scale plane stack, reordering the values of the original one. We then calculate a second distance image, reversing the swap by reinterpreting indices to be in line with the original distance image, and calculate the element-wise average. The work of Möller^43^ shows that this method is able to reduce homing errors, especially for challenging lighting conditions.

Note that we do not use coarse-to-fine search or early stopping during minimum calculation; both extensions suggested in the work of Möller et al.^2^.

Following Möller et al.^3^, we preprocess each input image $\varvec{I}$ by convolving it with a $2\times 1$ filter kernel as a basic measure of illumination tolerance:

8 $$\begin{aligned} \frac{\partial f}{\partial y} = \begin{bmatrix} -1 \\ +1 \end{bmatrix} * \varvec{I} \end{aligned}$$

This reduces the image height and the vertical position of the horizon by 1 pixel, measured from the top of the image. When we refer to MinWarping in the following, it always includes edge filtering, except when network preprocessing is used.

## Network-based image preprocessing

**Figure 6:**
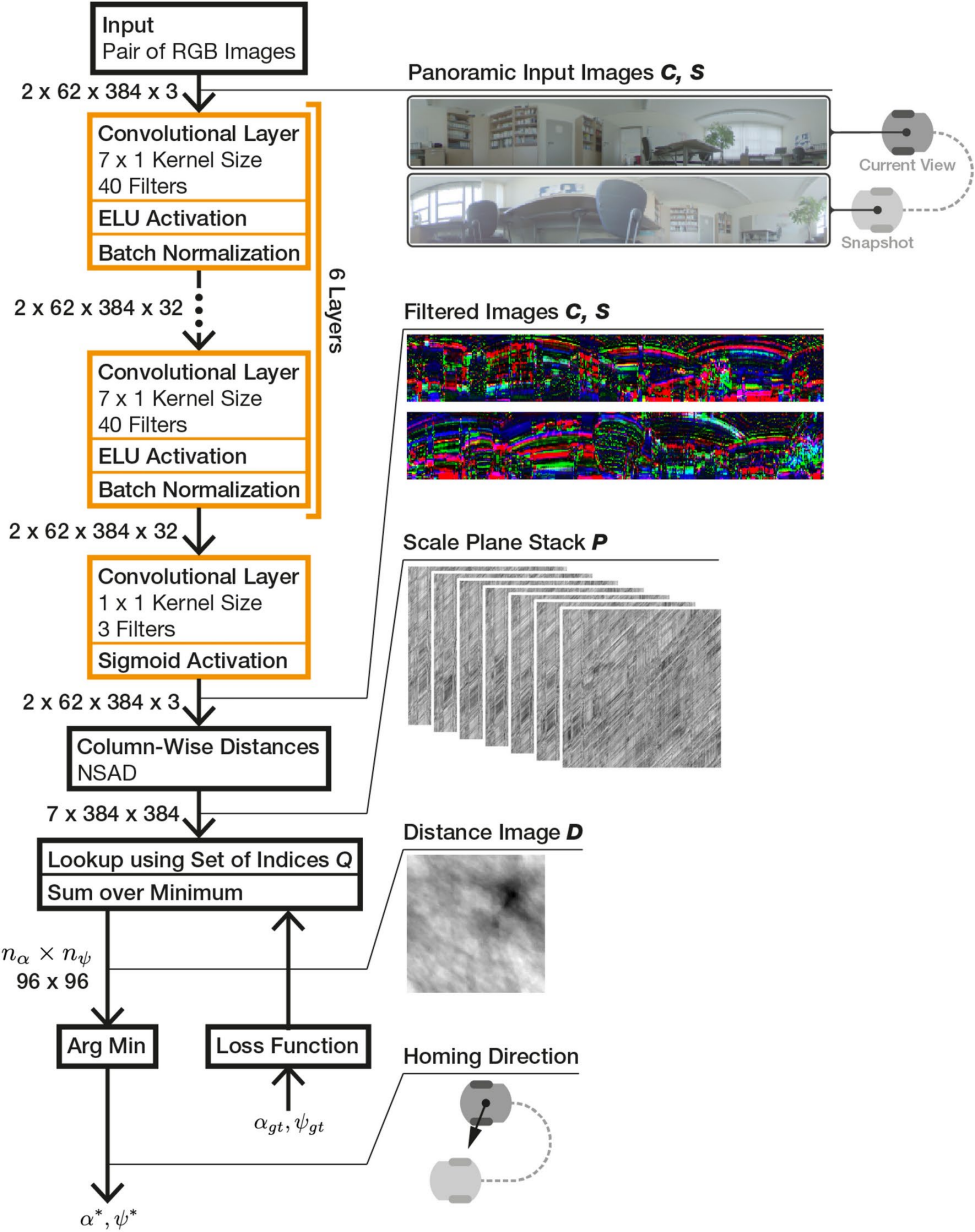
Overview of the proposed neural network (left, orange) embedded in the homing architecture (left, black) and computation results (right). The example images on the right are captured from an actual forward pass through the network and homing algorithm. The input images are selected from two different illumination variants of the setting Uni Office. Size annotations on the left side are in reference to panoramic color images ($2 \times \text {height} \times \text {width} \times \text {color channels}$) used throughout this work. For more information on the input images, see “Dataset”.

We extend MinWarping with a convolutional neural network to independently process the snapshot and the current view. It outputs a feature map with the same dimensions as the input images, which we use to estimate movement parameters with MinWarping. The combined algorithm with network parameters is shown in Figure 6.

To train the weights, we define a loss function (see “Loss function”) using the distance image $\varvec{D}$ from Eq. 7. Backpropagation through MinWarping involves computing the gradient of two chained functions: the sum over minima for all $i(\alpha ), i(\psi )$ (Eq. 7), and the column distance function $d(\varvec{A}, \varvec{B})$ (Eq. 4). Rescaling using nearest neighbor interpolation (“see Phase 1: Column-wise distances”) and the application of the set of indices $Q$ (“see Phase 2: Search for optimal movement parameters”) are look-up operations and do not require differentiation. Implementing MinWarping along with the neural network using the machine learning framework Tensorflow 2.12^44^ allows us to use automatic differentiation to obtain the gradient. In the following, we describe the network architecture, the training scheme, and the loss function.

### Network structure

We design the neural network to process RGB color images with 384 columns, 62 rows, and values in the range of [0,1] (see “Preprocessing”). It consists of 7 convolutional layers^45^; the first 6 use 40 vertical ($7 \times 1$) filters with ELU activation^46^ and the last layer reduces the number of channels from 40 to 3 using a $1 \times 1$ kernel (also described as a multi-layer perceptron with column-wise application by Lin et al.^47^). The network output is restricted to the original range [0,1] using a sigmoid function. The initial weights of the convolutional layers are set using the uniform Xavier initialization^48^ and biases are initialized with zeros. We use batch normalization^49^ after each of the first 6 layers with the default parameters given by Tensorflow 2.12^44^ (momentum = 0.99, $\epsilon = 0.001$, and both learned factors $\beta , \gamma$ enabled).

We retain the height of the input tensor for every convolutional layer by applying padding to the top and bottom edge and using a stride of 1 for the application of filters. We choose an axis reflection of values along the top or bottom row, as it offers the lowest average angular error with respect to the homing angle $\beta$ on the validation dataset (see Section S2 in the Supplementary Information).

In MinWarping, every image column is an individual landmark. Consequently, we choose convolutional kernels with a width of 1, meaning they are applied independently to each image column.

We also evaluated wider kernels, but the homing quality on the validation dataset did not improve. Note that for wider kernels, the cyclic nature of the panoramic input images has to be considered, which we realized by additionally padding input images for each convolutional layer along the horizontal axes by expanding the input image with itself at the left and right edge.

### Training scheme

Given the split of the dataset into disjoint training, validation, and test datasets, we are training the network using the Adam optimizer^50^ with a learning rate of 0.001 for at most 100 epochs. Any epoch contains 400 batches from the training dataset with 8 samples each. A sample is an image pair alongside ground truth movement information. To prevent overfitting, we calculate the average angular error with respect to the homing angle $\beta$ on 50 batches from the validation dataset and stop the training if the error did not decrease for 25 epochs, restoring weights from the last epoch with improvement.

### Loss function

**Figure 7:**
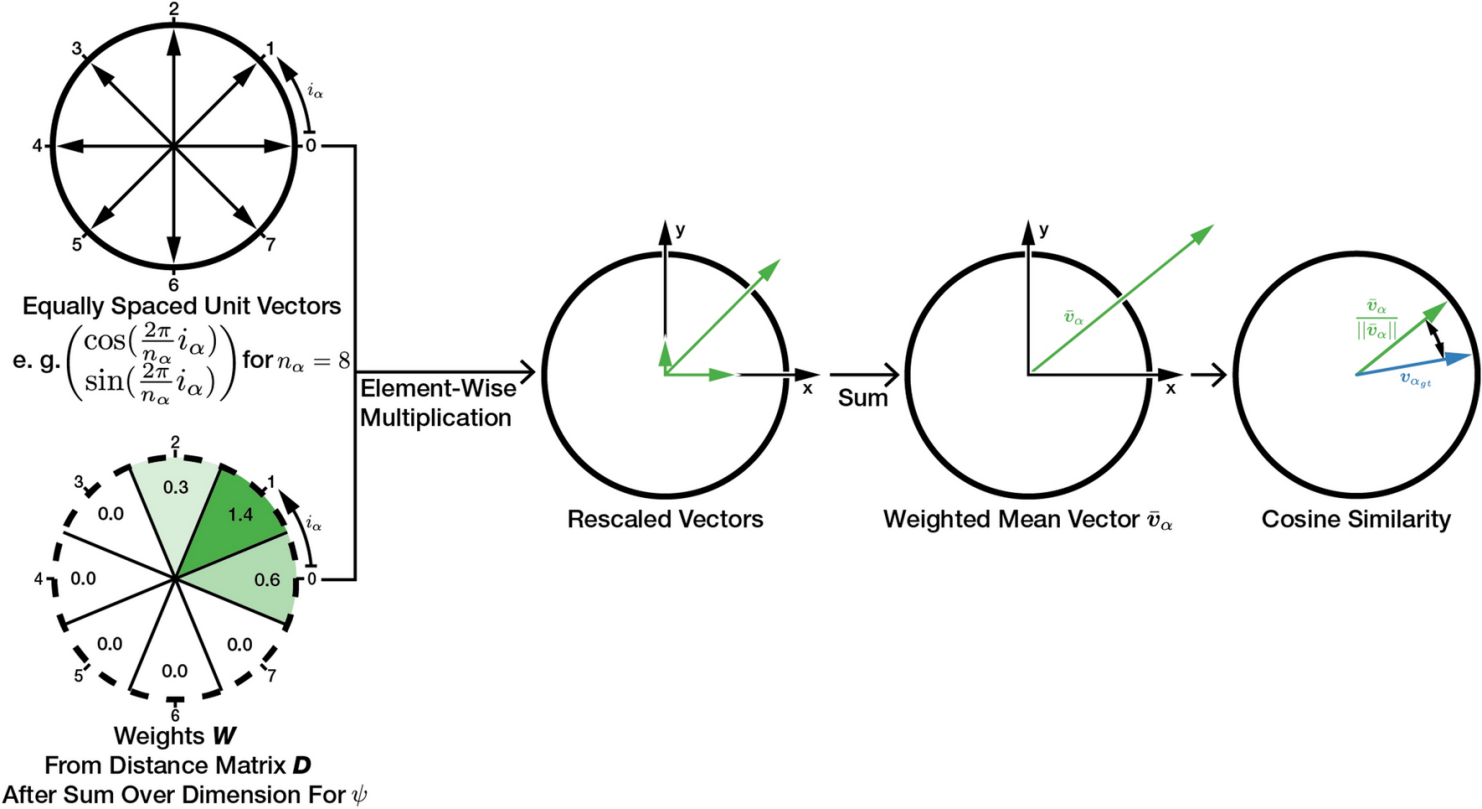
Example for the loss function computation up to the cosine similarity term for $\alpha$ and a step size $n_\alpha$ of 8. The 1D weights for $\alpha$ are computed by summation over the $\psi$-dimension of $\varvec{W}$. The solid circle marks the unit sphere and all prototype vectors have a length of 1. The dashed circle marks a cyclic array structure.

Learning to estimate movement parameters $\alpha , \psi$ (see “Overview of geometric constraints for 2D homing”) with the hybrid architecture is a regression problem. However, training with a loss function that uses optimal $\alpha ^*, \psi ^*$ estimated by MinWarping is unsuccessful: MinWarping selects the optimal movement parameters using an argmin function on the distance image $\varvec{D}$ (see Eq. 7), whose derivative is 0 everywhere but at the minimum. Therefore, gradients only propagate to the selected cell and the network cannot learn from the magnitude of errors in other cells. To avoid this, we create a smooth function to estimate the movement parameters $\alpha , \psi$ by reinterpreting $\varvec{D}$ as a weight matrix $\varvec{W}$ and computing a weighted mean over the angles $\alpha , \psi$ associated with each cell in $\varvec{D}$. To avoid the discontinuity in the scalar angle representation, we use a unit vector to represent both $\alpha$ and $\psi$ for every cell. We assume that both angles are independent and compute weighted means separately. An example for $\alpha$ is shown in Figure 7.

The weight matrix $\varvec{W}$ is obtained by inverting values in $\varvec{D}$ as follows, with $||\varvec{D}||_{\max }$ as the maximum norm and $\varvec{J}$ as an all-ones matrix:

9 $$\begin{aligned} \varvec{W} = \varvec{J}||\varvec{D}||_{\max }-\varvec{D} \end{aligned}$$

Values in $\varvec{W}$ are larger or equal to zero and the minimum in $\varvec{D}$ becomes the maximum in $\varvec{W}$. Using $\varvec{W}$, we calculate weighted mean vectors $\bar{\varvec{v}}_\alpha$ and $\bar{\varvec{v}}_\psi$. To obtain the 1D weights for $\bar{\varvec{v}}_\alpha$, we sum up elements in $\varvec{W}$ along the dimension for $\psi$, and vice-versa:

10 $$\begin{aligned} \bar{\varvec{v}}_\alpha = \sum _{i_\alpha =1}^{n_\alpha }\left( \begin{bmatrix}\cos \left( \frac{2\pi }{n_\alpha }i_\alpha \right) \\ \sin \left( \frac{2\pi }{n_\alpha }i_\alpha \right) \end{bmatrix}\cdot \left( \sum _{i_\psi =1}^{n_\psi }\varvec{W} _{i_\psi ,i_\alpha }\right) \right) \nonumber \\ \bar{\varvec{v}}_\psi = \sum _{i_\psi =1}^{n_\psi }\left( \begin{bmatrix}\cos \left( \frac{2\pi }{n_\psi }i_\psi \right) \\ \sin \left( \frac{2\pi }{n_\psi }i_\psi \right) \end{bmatrix}\cdot \left( \sum _{i_\alpha =1}^{n_\alpha }\varvec{W} _{i_\psi ,i_\alpha }\right) \right) \end{aligned}$$

Here, $n_\alpha ,n_\psi$ are the number of steps for the grid search described in “Phase 2: Search for optimal movement parameters” and the dimension of the distance image $\varvec{D}$ as well as the weight matrix $\varvec{W}$. A pair of values of $i_\alpha , i_\psi$ corresponds to an instance of the grid search and are indices into $\varvec{W}$.

We then determine the error between $\bar{\varvec{v}}_\alpha$ and the vector $$\varvec{v}_{\alpha _{gt}}=\begin{pmatrix}\cos (\alpha _{gt})\\ \sin (\alpha _{gt})\end{pmatrix}$$, derived from the ground truth angle $\alpha _{gt}$, using the negative cosine similarity

11 $$\begin{aligned} D_C(\bar{\varvec{v}}_\alpha , \varvec{v}_{\alpha _{gt}}) = -\frac{\bar{\varvec{v}}_\alpha }{||\bar{\varvec{v}}_\alpha ||} \cdot \varvec{v}_{\alpha _{gt}}. \end{aligned}$$

With an equally derived negative cosine similarity for $\psi$, the final loss function becomes

12 $$\begin{aligned} E(\bar{\varvec{v}}_\alpha , \varvec{v}_{\alpha _{gt}}, \bar{\varvec{v}}_\psi , \varvec{v}_{\psi _{gt}}) = D_C(\bar{\varvec{v}}_\alpha , \varvec{v}_{\alpha _{gt}}) + D_C(\bar{\varvec{v}}_\psi , \varvec{v}_{\psi _{gt}}). \end{aligned}$$

The proposed approximation is close to the argmin function if there is exactly one distinct minimum in $\varvec{D}$. If, in contrast, all values in $\varvec{D}$ are close, the directions of opposing unit vectors cancel each other out. In this case, the length of $\bar{\varvec{v}}$ approaches 0 and its direction is increasingly influenced by noise in $\varvec{D}$. As a result, the estimated movement parameters and the calculated loss are determined by a random process and training becomes impossible. This problem can occur if the image columns of the network output are indiscriminable, for example due to the choice of network architecture or the weight initialization. Choosing a purely convolutional neural network avoids this, because even randomly initialized CNN layers can offer decent image filtering quality^51^.

## Indirect relative pose estimation baseline

Besides MinWarping with edge filtering, we compare our proposed method with a pipeline for relative pose estimation using indirect methods. We follow Lindenberger et al.^4^, combining deep-learning based methods for feature detection, description (SuperPoint^16^) and matching (LightGlue^4^) with outlier rejection using RANSAC^10^. For SuperPoint and LightGlue, we use the reference implementation from Lindenberger et al.^4^ and use the network weights supplied by the authors (https://github.com/cvg/LightGlue/releases/tag/v0.1_arxiv). The maximum number of keypoints for SuperPoint is set to 2048.

We supply the SuperPoint network with the same panoramic image format used for the other methods in this work.

From the matches provided by LightGlue, we determine the relative camera pose using epipolar geometry with a planar motion assumption using the two-point algorithm “2pt_circle” from Choi and Kim^52^. Exploiting the additional constraint for the given application of indoor robots with 2D movement leads to faster computation and better homing quality when compared to a general 5D solution using five matches^53^.

We use the two-point algorithm as part of a RANSAC implementation. Hereby, we follow the library of Kneip and Furgale^54^ and first transform image points to normalized bearing vectors $\varvec{f}$ in each camera frame. Then, we determine a set of essential matrices from two randomly selected matches using the two-point algorithm and use the remaining matches to select the essential matrix with the least reprojection error. For the best essential matrix, we calculate the number of inliers by reprojecting points from $\varvec{S}$ and $\varvec{C}$ to the respective other camera. We use the geodesic reprojection error $1-\varvec{f}_{\text {meas}}\cdot \varvec{f}_{\text {repr}}$ based on the cosine of the angle between the normalized bearing vectors for measurement $\varvec{f}_{\text {meas}}$ and reprojection $\varvec{f}_{\text {repr}}$ as suggested by Kneip and Furgale^54^. The inlier threshold is computed as $t = 1-\cos (\theta _{\text {threshold}})$. Considering the image width of 384 px used throughout this work, we select $\theta _\text {threshold} = 1^{\circ }$, which amounts to $\sim$1 px error. We add reprojection errors for both cameras, any matches with errors below $2t$ are counted as inliers. Like Kneip and Furgale^54^, we terminate the RANSAC algorithm after $k = \frac{\log (1-p)}{\log (1-w^n)}$ steps, but after at most 500 iterations. Hereby, $w$ is the inlier ratio, $p$ the probability that algorithm has selected a set with only inliers, and $n$ the number of drawn samples. The inlier ratio $w$ is determined at runtime using the currently found best model. We select $p=0.999$. Both $p$ and the maximum number of steps are manually selected using situations with few inliers due to large baselines and without regarding execution time.

## Metrics

We use two metrics to quantify homing quality: the average angular error (AAE) and the return ratio (RR).

### Average angular error (AAE)

The Average Angular Error (AAE) is used as a general measure of homing quality. The smaller the AAE, the more likely the homing succeeds and the straighter the homing paths. Given a batch of size $n$ with snapshot and current view images and associated ground truth homing angles $\varvec{\beta }_{gt}$, the AAE of the homing results $\varvec{\beta }$ is computed as follows:

13 $$\begin{aligned} \text {AAE}(\varvec{\beta }_i, \varvec{\beta }_{gt,i}) = \frac{1}{n}\sum _i^n \arccos (\cos (\varvec{\beta }_i-\varvec{\beta }_{gt,i})) \end{aligned}$$

By applying the cosine and inverse cosine functions, we are only considering the shortest angular difference between $\varvec{\beta }$ and $\varvec{\beta }_{gt}$, meaning an angle of 180° is the largest possible homing error for a single image pair. Any future use of the AAE refers to the homing angle $\beta$.

We calculate the AAE for a setting two ways: First, the AAE over all possible image pairs (excluding images at the same grid position) serves as an indicator of the general homing quality.

Second, we group image pairs by spatial distance and assess the AAE within each bin. We expect the homing to be more difficult for shortest and longest distances between snapshot and current view. When images become increasingly similar due to small distance between capture positions, the landmark movement in the image becomes minimal and the influence of noise increases. The farther snapshot and current view positions are apart, the more likely it is that landmarks get occluded, reducing the number of columns that can be successfully matched. Examining the AAE by distance allows a quantification of this effect. Note that the relative pose of a camera can only be determined up-to-scale when monocular images are the only data source. This is because the distance between snapshot and current view depends on the distance of each camera pose to the landmarks (see Figure 4), and no absolute measurement is given. Therefore, the evaluation by camera distance presented in this work is only valid in reference to the average scale of the tested home and office environments.

### Return ratio (RR) and inverse RR based on the homing vector field

It is common practice to recompute homing angles multiple times while approaching the snapshot, compensating for errors both in the homing process and in the robot control. Therefore, the angular error becomes less meaningful to the success of the homing attempt as the spatial distance between the snapshot and current view gets larger. We simulate homing with multiple steps on the grid of images for any given snapshot and calculate the fraction of successful homing attempts as the return ratio (RR) described by Möller et al.^2^.

We compute the RR for a setting considering all possible snapshot positions and variant combinations, including constant illumination. For every variant pair, we generate a homing vector field by choosing a snapshot position from the first variant and computing homing vectors from every current view of the second variant. We also use this intermediate result for further analysis in the section “Inverse return ratio (IRR)”. Next, we simulate pathfinding from every current view position to the snapshot by moving to the adjacent grid cell the home vector points towards. For this, we consider grid indices rather than ground truth positions and discretize the homing angle to 45° increments. A homing attempt fails if the simulated agent leaves the grid or the snapshot position is not reached within a predefined number of steps, which we select as the sum of the outer dimensions of the grid. The total return ratio for a setting is computed by averaging over every snapshot position and variant pair combination.

Because the RR for all investigated methods is high, we show the inverse return ratio ($1-\text {RR}$; referred to as IRR) for better visibility. The IRR conforms to the fraction of failed return attempts.

## Results

We first assess the homing quality for color, HDR imaging, and histogram equalization for MinWarping with edge filtering. The subsequent evaluation of the network consists of two parts: a comparison with competing methods using AAE and RR and an analysis of the network output.

### Selecting the optimal image preprocessing using MinWarping with edge filtering

**Figure 8:**
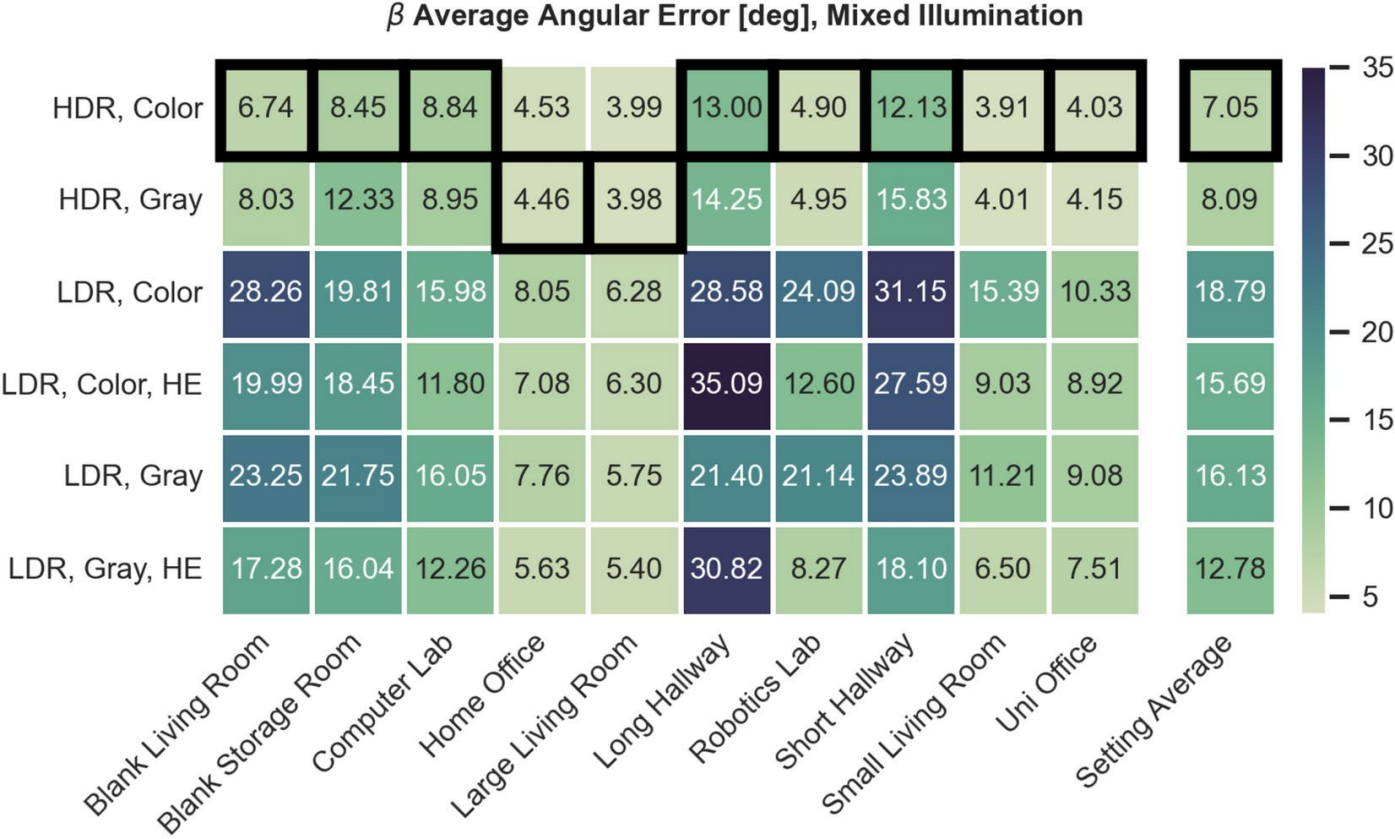
AAE for MinWarping with edge filtering and different image preprocessing methods. Illumination between snapshot and current view is mixed. Results are shown in degrees. The best results for each setting are marked with a black border.

**Figure 9:**
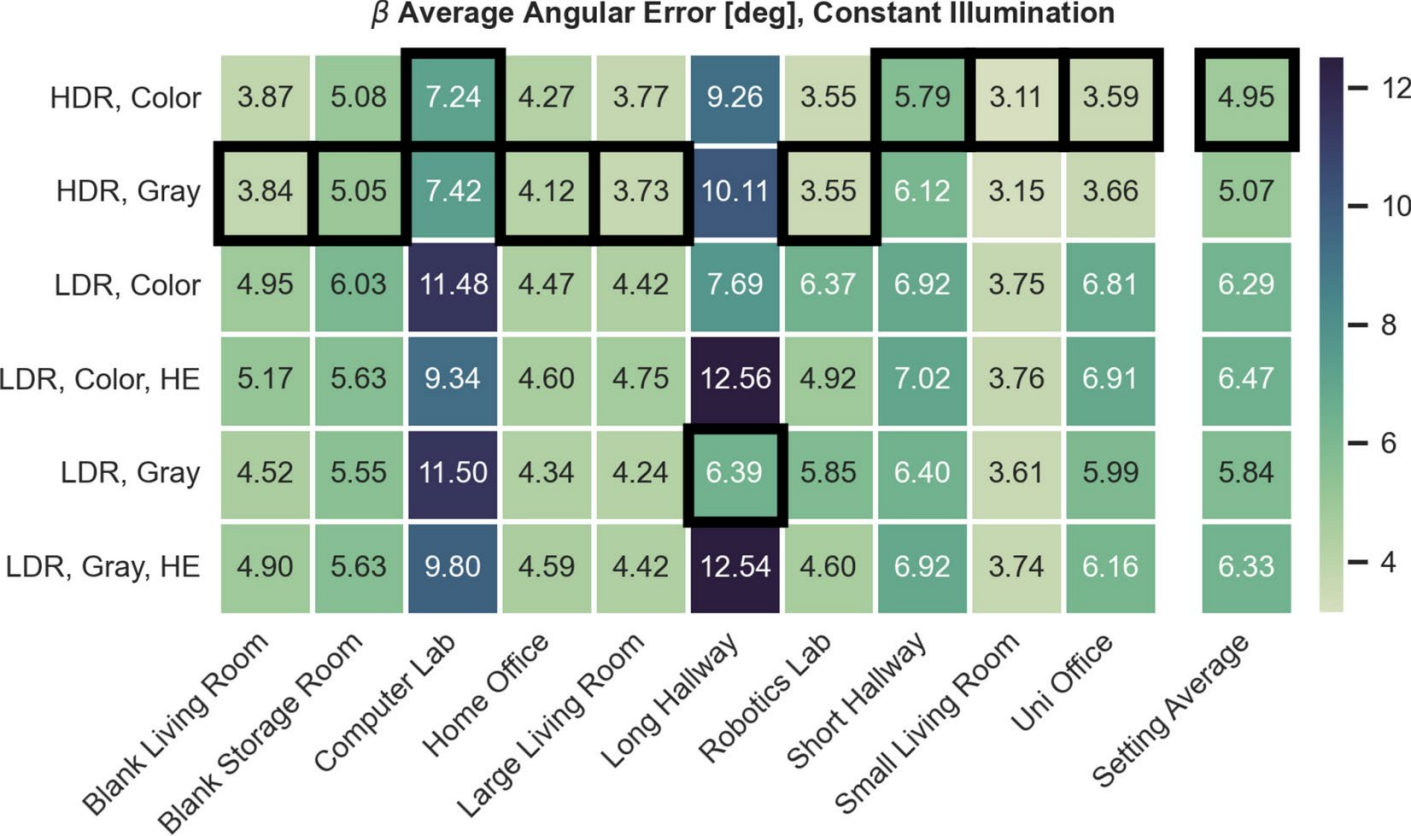
AAE for MinWarping with edge filtering and different image preprocessing methods. Illumination between snapshot and current view is constant. Results are shown in degrees.

Previous work on MinWarping uses grayscale images with histogram equalization and an exposure time controller for indoor scenes^53,55^. Meanwhile, HDR images with color information are the superior choice for outdoor environments when considering indirect navigation methods^56^. To select optimal image inputs for model training and ensure competitiveness of MinWarping with edge filtering, we test the usage of HDR, LDR, color, and histogram equalization. For LDR images, we emulate a lighting controller by selecting the image from the exposure series with the mean gray value closest to 40 considering an 8-bit range. For every setting, we select all available image pairs, excluding same positions, and compute the AAE. The dataset averages are computed as a mean of the setting-wise results. The AAE for mixed illumination is presented in Figure 8 and results for constant illumination are shown in Figure 9.

Using HDR instead of the best LDR type for mixed illumination, grayscale with histogram equalization, reduces the dataset-wide AAE by $\sim$30%. When only LDR images are available, histogram equalization can improve homing results under illumination changes for all tested settings except Long Hallway. Using color information for LDR images increases homing error as well as computation time and therefore offers no additional benefit.

The lowest AAE across settings is achieved using HDR color images for both constant and mixed illumination. Comparing homing errors for individual settings, using HDR with color information is consistently comparable or better than the next best preprocessing variant, grayscale HDR, and is therefore selected for network training and all evaluation tasks, including the indirect RPE pipeline.

### Homing quality comparison

We evaluate homing quality differences between MinWarping with network-based image preprocessing, MinWarping with edge filtering, and the indirect relative pose estimation (RPE) pipeline. We also compare the homing quality for mixed and constant illumination pairs.

#### Average angular error (AAE) by setting

**Figure 10:**
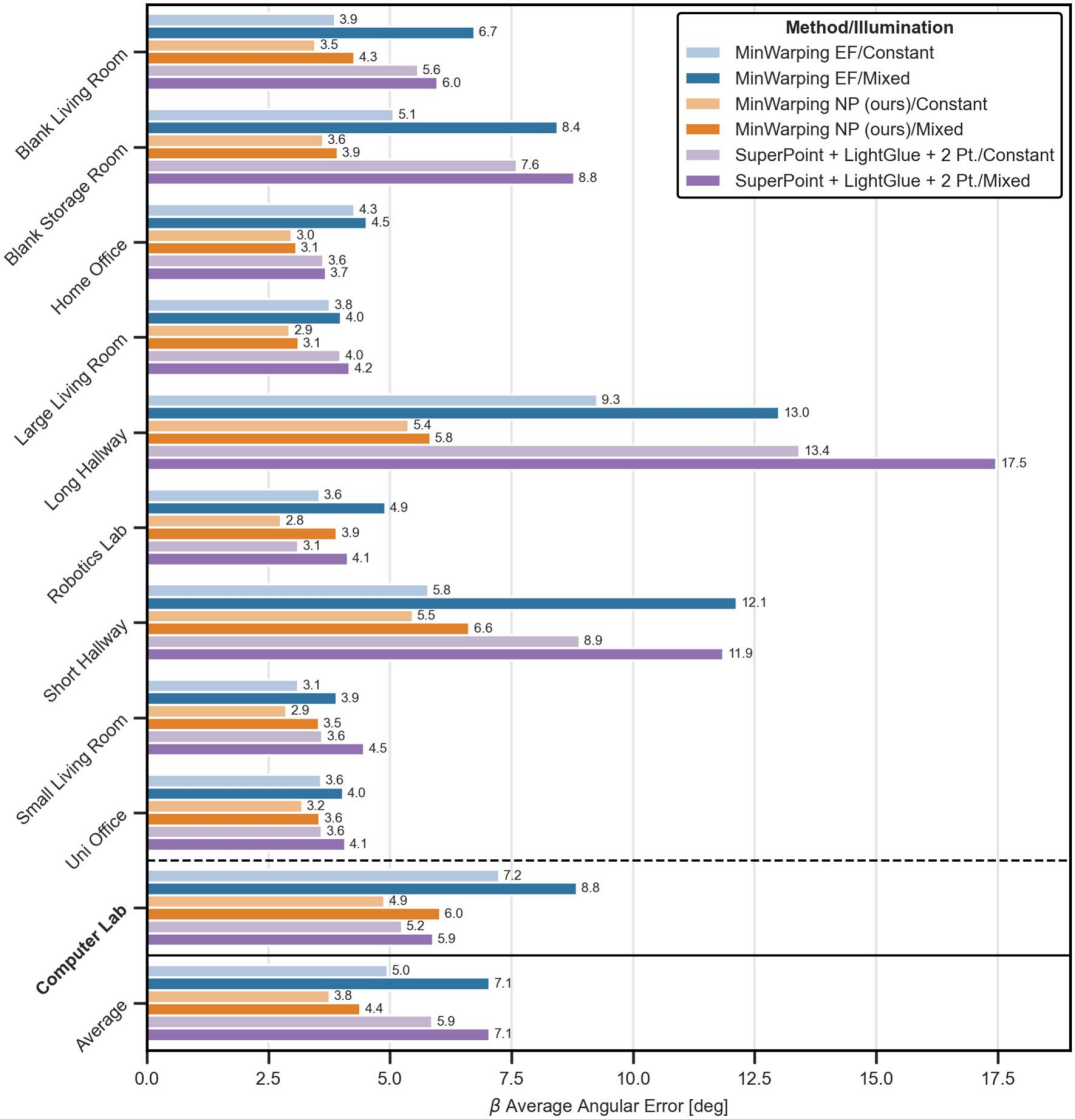
Setting-wise Average Angular Errors (AAEs) of $\beta$ homing angle in degrees. The group at the bottom shows averages for the whole dataset. Results are color coded as follows: *blue:* MinWarping with edge filgering (MinWarping EF), *orange:* homing with network-based preprocessing (MinWarping NP), *violet:* indirect RPE pipeline, *light color:* constant illumination, *dark color:* mixed illumination. Results are computed as described in Section Average angular error (AAE). Every bar is annotated with the AAE rounded to 1 decimal place. Dataset-wide averages (bottom) are the mean over the per-setting averages. The setting Computer Lab (bold) is used as a disjoint test dataset and was not used during training or validation.

As shown in Figure 10, MinWarping with network-based image preprocessing (“MinWarping NP”) generally provides the lowest AAE out of the three compared methods. In relation to MinWarping with edge filtering (“MinWarping EF”), the AAE is reduced for every setting and on average by $\sim$38% from 7.1° to 4.4° for mixed illumination and by 24% from 5.0° to 3.8° for constant illumination. The quality improvements generalize to the test setting Computer Lab, for which MinWarping NP reduces the AAE by $\sim$32% from 8.8° to 6.0° for mixed illumination and by $\sim$32% from 7.2° to 4.9° for constant illumination. The AAE reductions for constant illumination indicate that network-based preprocessing is in general beneficial for the homing task. Additionally, it reduces the gap in AAE between situations with constant illumination and those with mixed illumination: the AAE increases by $\sim$42% for EF and by $\sim$16% for NP.

MinWarping EF and the indirect RPE pipeline offer a similar homing quality, except for Computer Lab, where the indirect RPE pipeline is on par with MinWarping NP. Notably, for $\sim$0.05% of image pairs, the number of feature matches provided by LightGlue was too low to fit the two-point model, for example in situations with long baselines, sparse features and strong illumination changes. These cases contribute to the average of the indirect RPE pipeline with a homing error of 90°.

**Figure 11:**
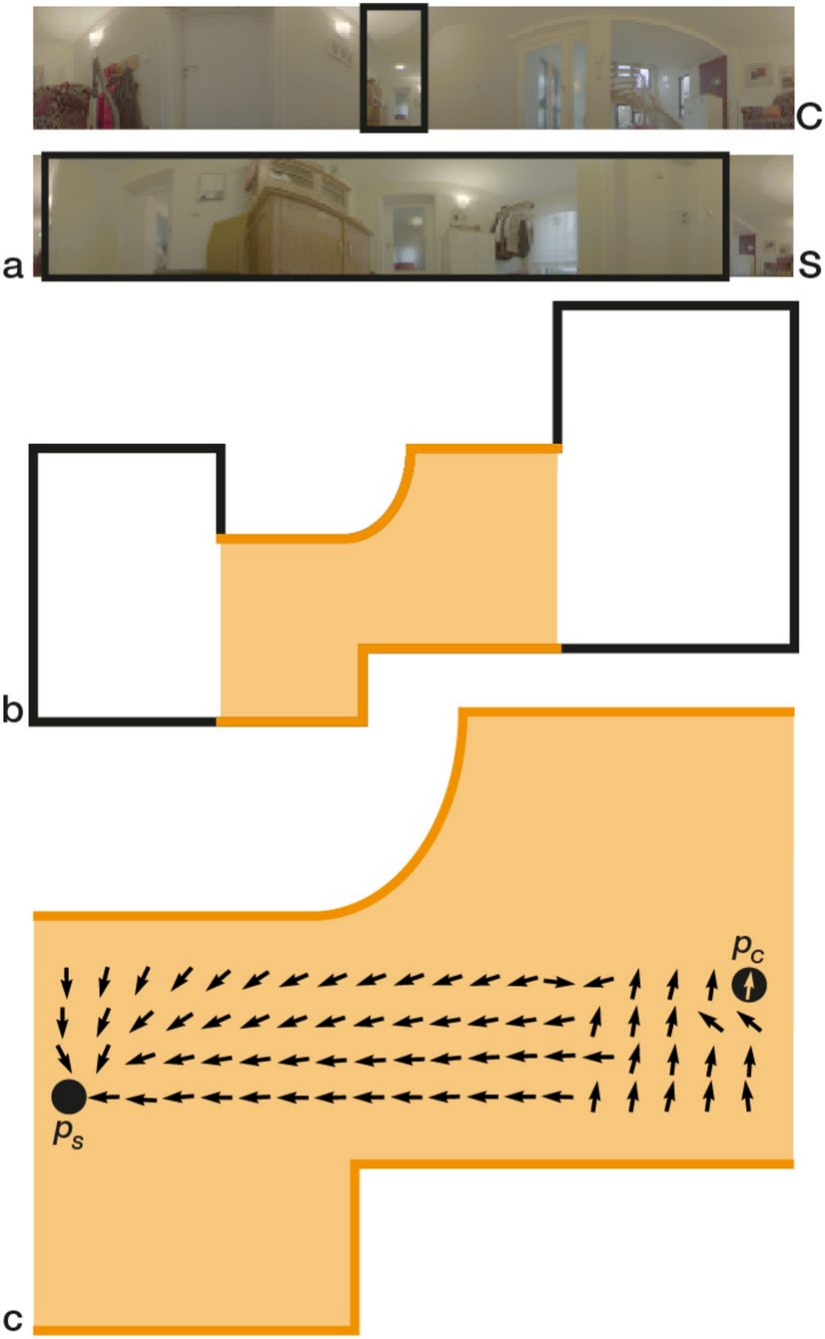
Depiction of the room geometry for the *Long Hallway* setting and its impact on homing quality. Part **a** shows images for a snapshot image S and a current view image C at the positions $\varvec{p_S}$ and $\varvec{p_C}$. Black boxes mark the the same image region for $\varvec{S}$ and $\varvec{C}$ in the vicinity of the FOC. Part **b** is a schematic of the room geometry for Long Hallway. The capture region is marked in orange. Part **c** shows an exemplary homing vector field for the given snapshot and constant illumination.

The homing quality for Long Hallway with constant illumination is noticeably worse than for other settings. To investigate, we compute a homing vector field for the snapshot position at grid index (0,0) with MinWarping EF (see Figure 11). For a distance greater than about 2.4 m along the horizontal axis, home vectors point away from the snapshot, causing large angular errors. Example images show that walls limit the view of the open space at the opposite side of the mapped area due to the non-convex room geometry. In this situation, MinWarping NP reduces the AAE from 9.3° for MinWarping EF to 5.4°, indicating that the network finds a solution to better tolerate occlusion. This is a part of our network analysis in “Analysis of network output”.

**Figure 12:**
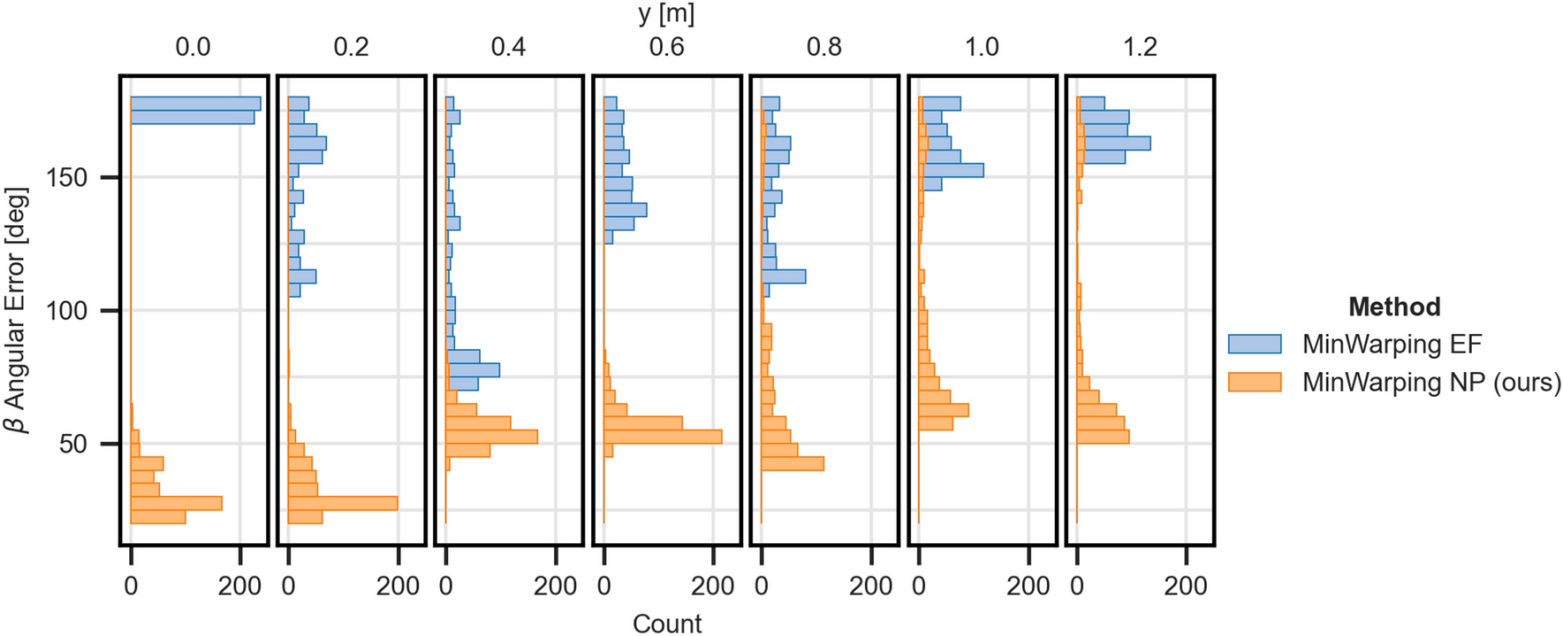
Histograms of angular errors above the 99th percentile of the Short Hallway setting for the methods MinWarping NP (orange) and MinWarping EF (blue) with mixed illumination. For each histogram, angular errors for a single grid point along the y-axis are grouped over grid points of the x-axis and variant pairs, excluding pairs with constant illumination. Positions near the origin are close to the wardrobe and coat rack, positions at about 1.2 m are close to the bare wall with the door (see Figure 2).

**Figure 13:**
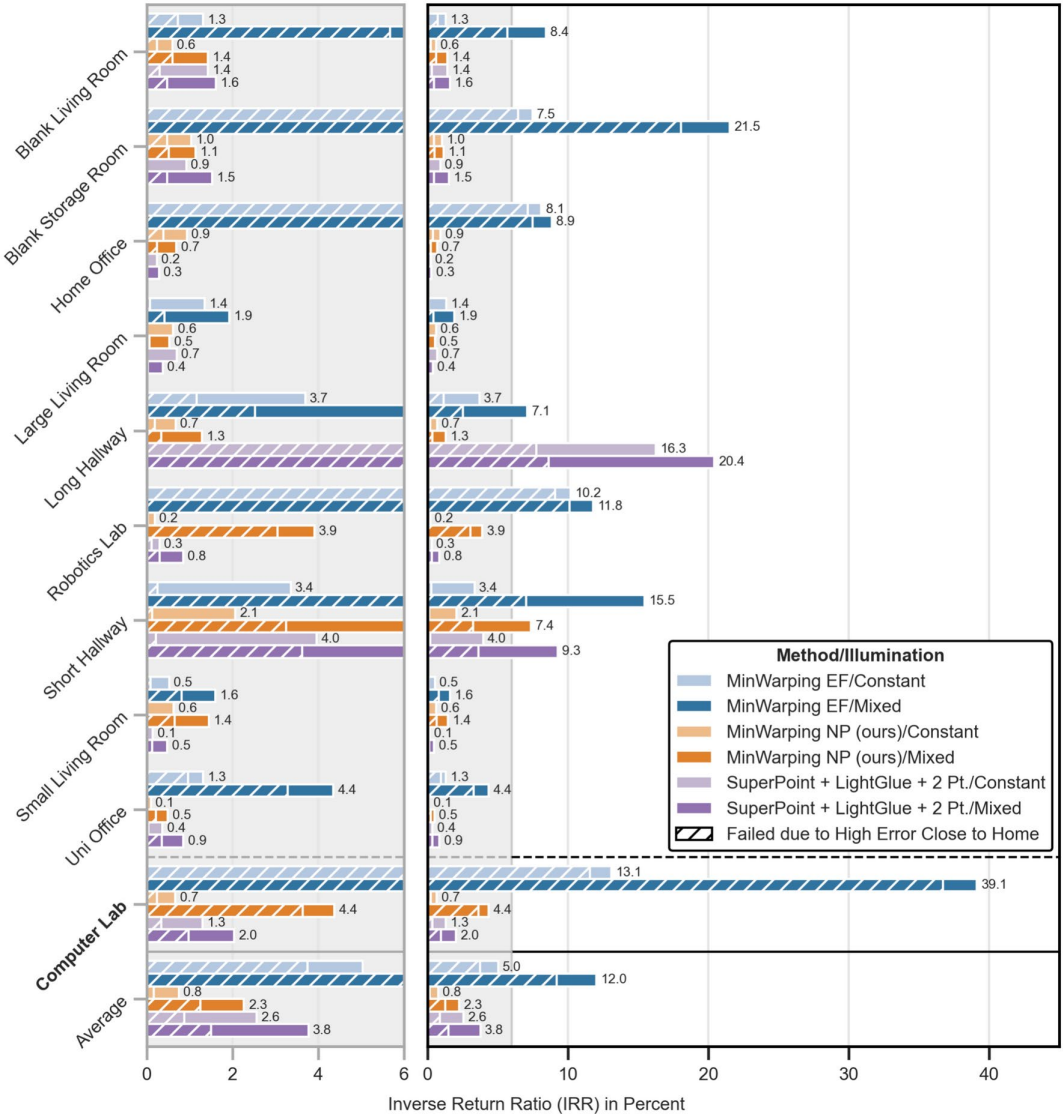
Setting-wise inverse return ratios (IRRs) as the percentage of failed return attempts. The group at the bottom shows averages for the whole dataset. Results are color coded as follows: *blue:* MinWarping with edge filgering (MinWarping EF), *orange:* homing with network-based preprocessing (MinWarping NP), *violet:* indirect RPE pipeline; *light color:* constant illumination within image pairs, *dark color:* mixed illumination. The fraction of return attempts that failed due to a high homing error close to the home position (“loops”, see Figure 14) are hatched. The unmarked fraction displays failures due to the simulated agent leaving the grid. Results are computed as described in Section “Average angular error (AAE)”. Every bar is annotated with the IRR in percent rounded to 1 decimal place. The right plot shows an overview, the left one a scaled view of the lower 6%. The scaled area is marked in gray. Dataset-wide averages (bottom) are the mean over the per-setting averages. The setting Computer Lab (bold) is used as a disjoint test dataset and was not used during training or validation.

To investigate the limitations of MinWarping NP, we look at the homing errors at individual grid positions for the setting with the worst homing quality, Short Hallway with mixed illumination. To this end, we gather the angular errors for homing from a selected grid position to any other position, considering all variant pairs with mixed illumination. We then group angular errors along the x-axis and select the upper 1% of the largest homing errors. As shown in Figure 12, there is a notable difference in the distribution of the errors along the y-axis of the room. For grid positions farther away from the origin, the distribution mass shifts towards larger errors, with a maximum of 180° for the last two grid locations. Hereby, positions near the origin are close to the two main objects in the room, the wardrobe and coat rack, and positions at about 1.2 m are close to a bare wall with a door (see Figure 2). We hypothesize that in settings with uneven distribution of visible objects, homing becomes harder if the distance to relevant landmarks increases. However, 99% of homing errors are smaller than the lowest shown histogram bin and even in the worst case, MinWarping NP provides a meaningful improvement over MinWarping EF.

#### Inverse return ratio (IRR)

Results for the inverse return ratio (IRR) are shown in Figure 13. We observe a clustering of results at and below 2%, a score that we regard as a low failure rate.

The IRR for MinWarping EF varies between settings: for constant illumination, we observe a low failure rate for 4 out of 10 settings, while for mixed illumination only 2 out of 10 settings qualify. The worst results are reported for the setting Computer Lab with 39.1% (mixed illumination) and 13.1% (constant illumination).

The IRR for the indirect RPE pipeline is on average only 1.5 points (constant illumination)/1.2 points (mixed illumination) higher than the scores for MinWarping NP. Both hallway settings pose problems for the indirect RPE pipeline, but it achieves a low failure rate for all other settings and exceeds the homing quality of both MinWarping methods in Computer Lab and Robotics Lab with mixed illumination, as well as Home Office and Small Living Room regardless of illumination.

MinWarping NP achieves a low failure rate for constant illumination for 9 settings. With mixed illumination, we report a low failure rate for 7 out of 10 settings, with Short Hallway showing the worst homing quality with 7.4% of failed return attempts. Besides Short Hallway, the settings Computer Lab and Robotics Lab also exhibit above-average failure rates.

**Figure 14:**
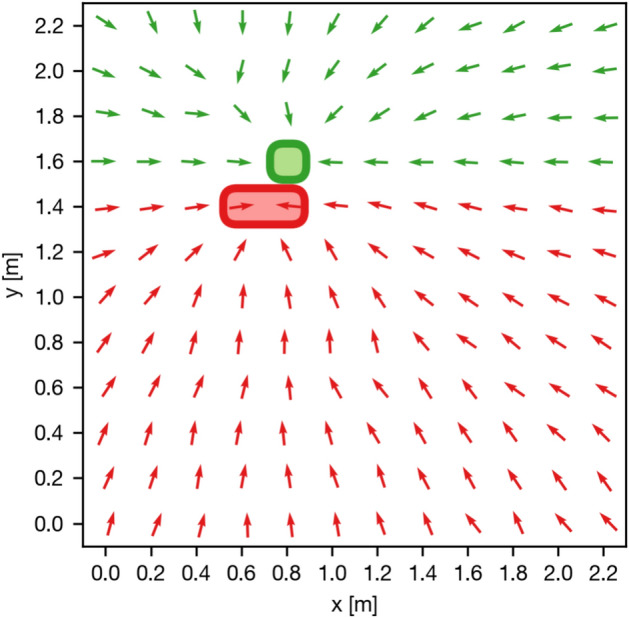
Example of a homing vector field for MinWarping NP, Computer Lab, and mixed illumination. The snapshot is located at (0.81 m, 1.6 m) and marked with a green box. We display homing directions as arrows at their recorded ground truth positions. Green arrows mark starting positions from which the simulated agent was able to reach the snapshot position while red arrows mark failed return attempts. The loop preventing a large percentage of runs to return to the snapshot is marked with a red box.

Investigating this, we discriminate the failed return attempts into two classes: either the agent left the grid or the maximum number of steps were exceeded. Following Figure 13, we find that exceeding the step limit is a main failure reason. In the example shown in Figure 14, we observe a large percentage of homing attempts ending in a loop due to high homing errors close to the snapshot position. Systematically detecting loops in homing vector fields for all combinations of settings and variants, we find that the median of the remaining distance is 1 grid cell regardless of illumination. Thus, even in cases in which the maximum number of steps during homing were exceeded, the robot often stops adjacent to the correct cell.

#### Average angular error (AAE) by distance

**Figure 15:**
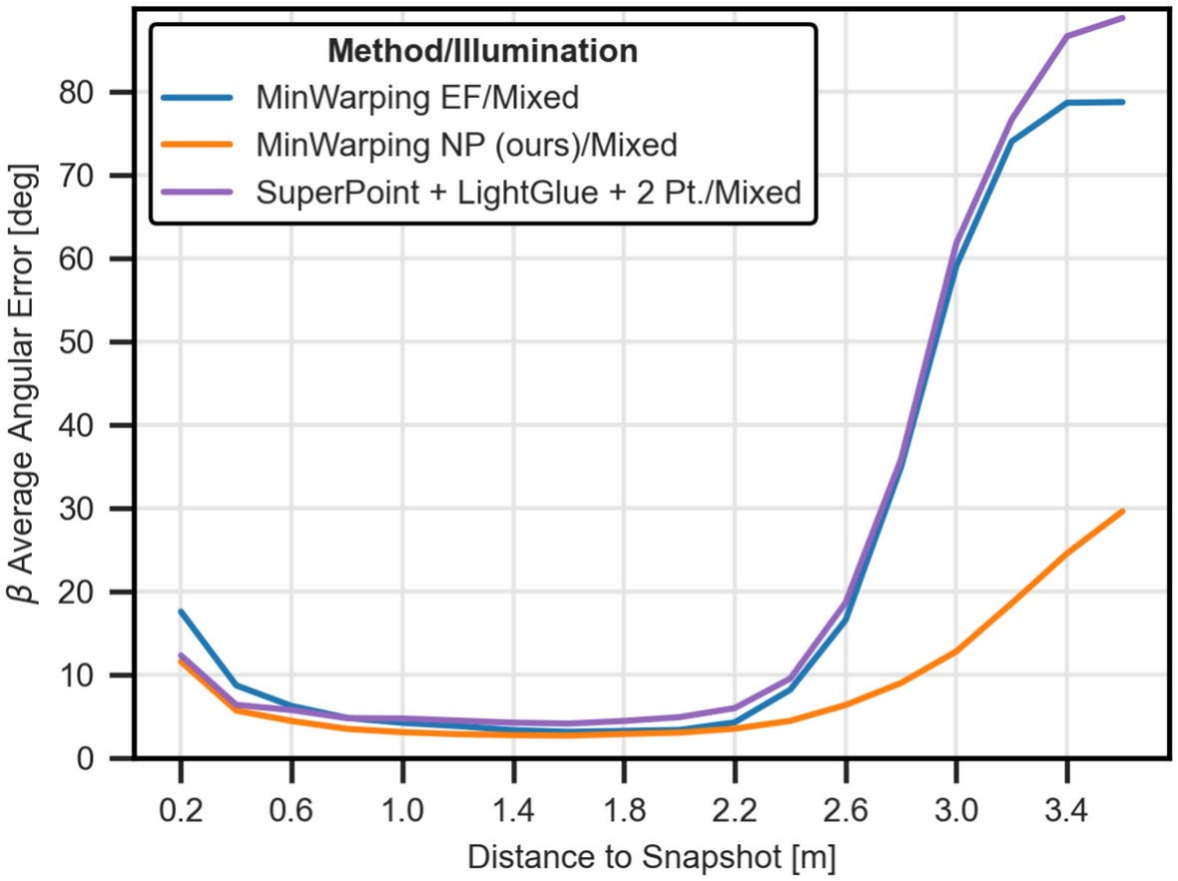
AAE of $\beta$ homing angle by distance between image capture locations. We compute results as described in the section “Average angular error (AAE)”. The homing error for the baseline is shown in blue, the error for homing with network preprocessing is shown in orange. Both results are based on mixed illumination.

In Figure 15, we examine the AAE by distance by sorting pairs of snapshot and current views with mixed illumination into bins of 20 cm width using the recorded positional information. Bin boundaries are equally spaced between 0.1 and 3.6 m. Distances beyond 3.0 m are solely contributed by the Long Hallway setting with partially occluded views (see “Average angular error (AAE) by setting”), so angular errors for these distances are likely exaggerated.

Using the network for image preprocessing improves the homing quality noticeably for far and short distances when compared to edge filtering. Both methods reach a similar minimum AAE at around 1.6 m. The indirect RPE pipeline offers similar homing quality to MinWarping EF, but improves upon distances below 0.8 m. For distances between snapshot and current view smaller than 1.6 m, the homing error gradually increases, particularly for current views adjacent to the snapshot (0.2 m). This result is in line with the partially poor return ratio due to loops close to the snapshot position.

### Analysis of network output

**Figure 16:**
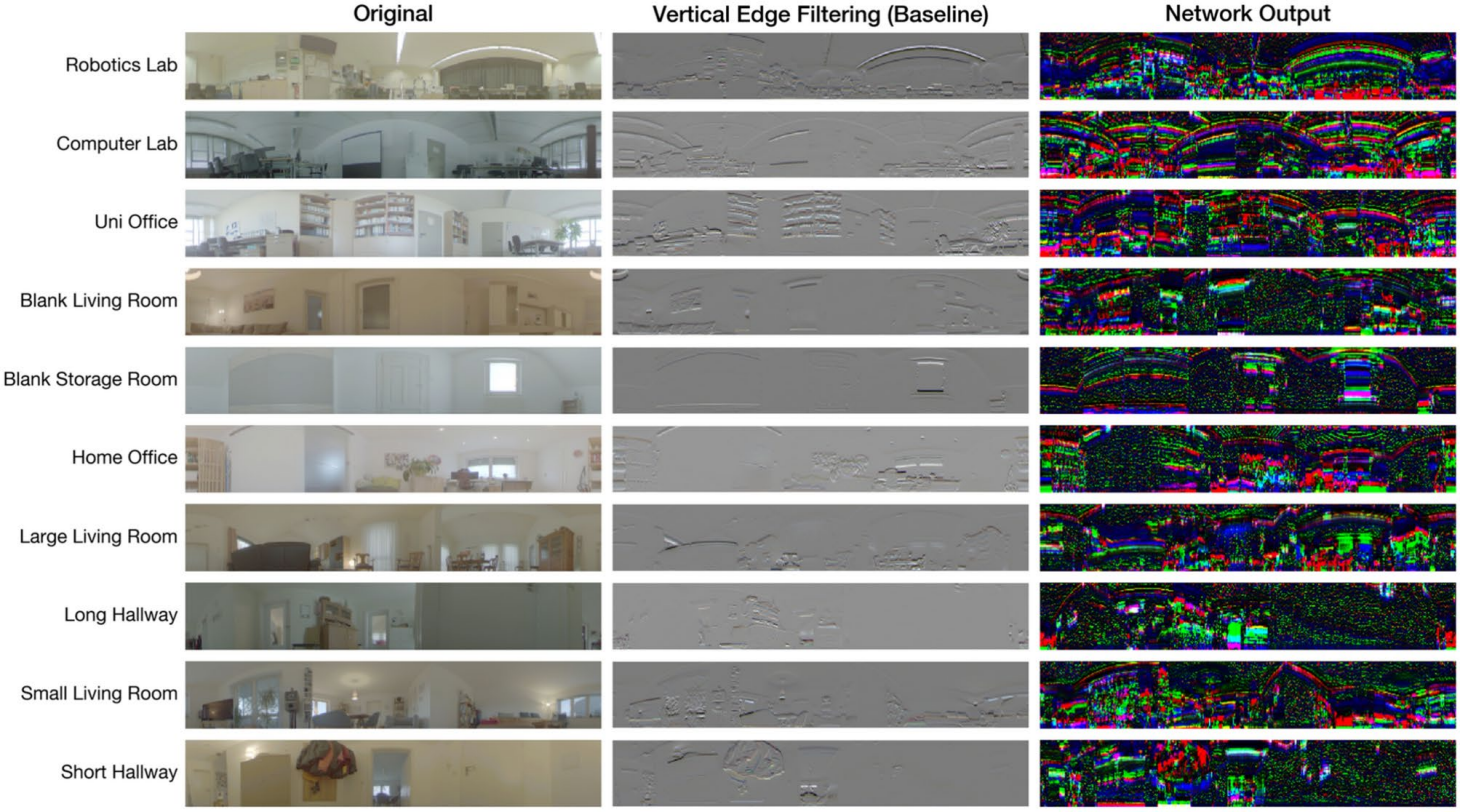
Comparison of example images for each setting. We present HDR images on the left, showing the state before edge filtering or application of the network. The middle column shows the same RGB images with vertical edge filtering applied as described in the section “Extensions to the MinWarping algorithm”. The RGB channels are jointly normalized to the range [0,1] (32-bit floating point) for better visibility. The right column shows the selected example images after network filtering is applied. The 3 output channels are interpreted as RGB colors in the range [0,1].

We compare the network outputs at the last layer to the original HDR color images and the effect of edge filtering in Figure 16.

The overall scene structure is still recognizable in the network outputs due to the topology-preserving property of CNNs, but the colors of the input images are replaced with strong responses to high image frequencies. This is similar to early convolutional layers of deep neural networks for image classification (see e. g. the work of Olah et al.^57^). Compared to edge filtering, which responds similarly to any of the input channels, activations of the network often show clear red, green, or blue colors and therefore overlap noticeably less. Furthermore, salient parts of the input signal are not only amplified, but also increase in size (e. g. wider edges of window frames, larger door handles). Finally, the network generates sharp noise for image regions with few details, particularly in the green channel.

**Figure 17:**
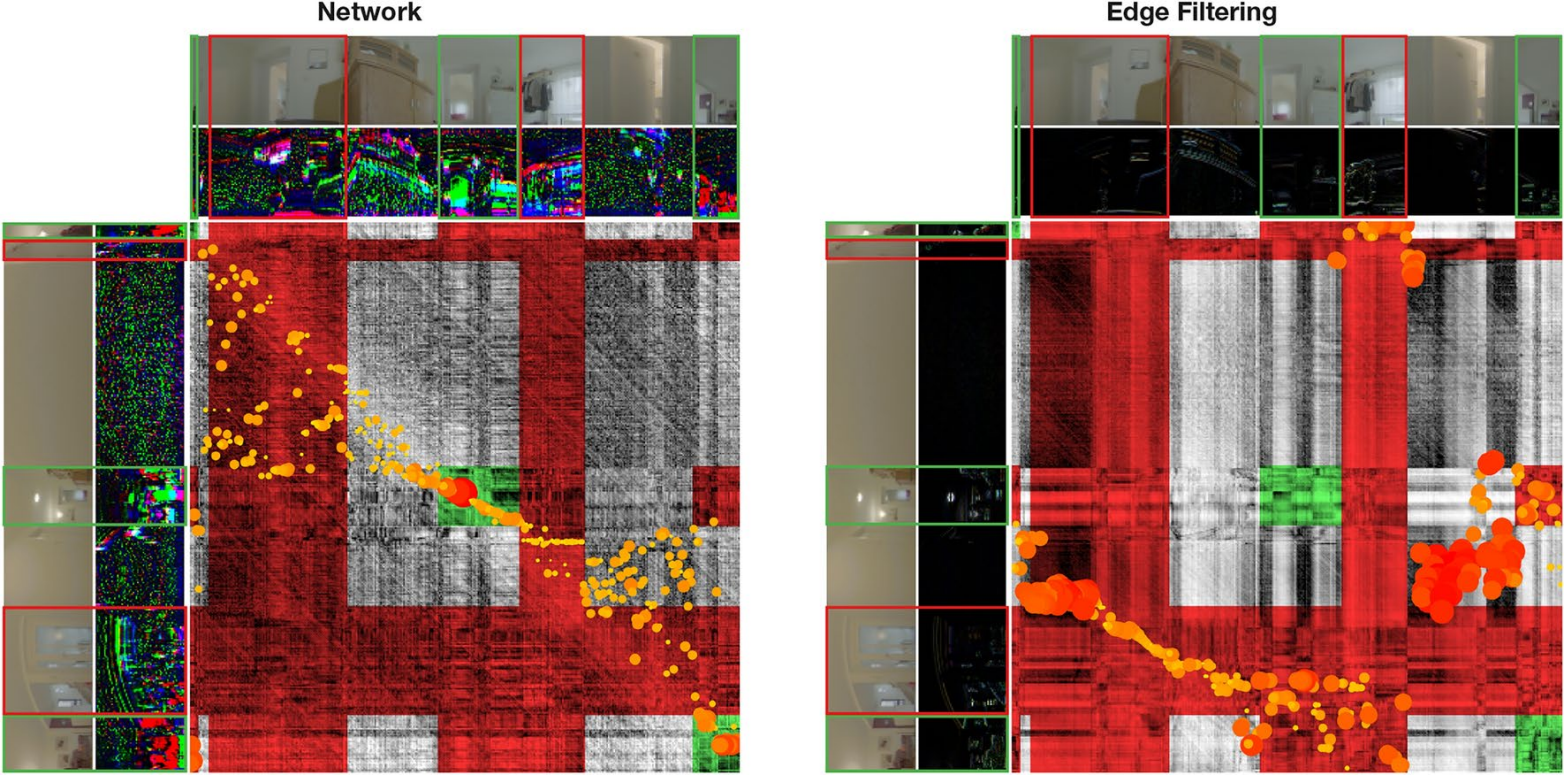
Searching columns with minimal distance in the scale plane stack in a situation with occlusion and image regions with few details (taken from the Long Hallway setting). For each subplot, we display a single scale plane with unscaled snapshot and current view ($\sigma = 1.0$). We remove the distortion of the scale plane defined by the index shift described in the section “Extensions to the MinWarping algorithm” such that each distance entry aligns with the columns of the images displayed on the edges. The images displayed on the side correspond to the snapshot (top) and current view (left). Both the original as well as the preprocessed images are shown. We mark visible regions with green boxes in the images and with a green tint in the scale plane. Red markers of images and scale plane show columns that are not visible and therefore cannot be matched between snapshot and current view. We display the position and importance of minima with an overlayed scatterplot. The importance of a minimum is calculated by normalizing all minima that contribute to the movement parameters estimated by MinWarping. The highest minima are displayed with small, yellow points; the lowest minima are represented with large, red points. Values in between interpolate simultaneously in size and color. The angular error for MinWarping NP is $\sim$7.73°, and for MinWarping EF $\sim$93.85°.

To analyze the effect of the network output on homing, we investigate the scale plane stack for an image pair from the Long Hallway setting in Figure 17. In the shown situation, MinWarping EF misestimates the homing angle by $\sim$93.85°. This is due to the algorithm having to match every column: with some columns occluded, the homing process must include mismatches. In this case, a correct result can only be achieved when the influence of correctly matched columns is dominant. In the situation shown in Figure 17, however, the low distances caused by columns with few details largely determine the result. These columns match well with each other, resulting in large regions with small distances in the scale plane of MinWarping EF.

The network generates noise for columns with few details, causing them to have a high distance to any other column. Simultaneously, columns with salient image features match well. In combination, MinWarping NP can better focus on parts of the scene that are beneficial to a correct homing result.

**Figure 18:**
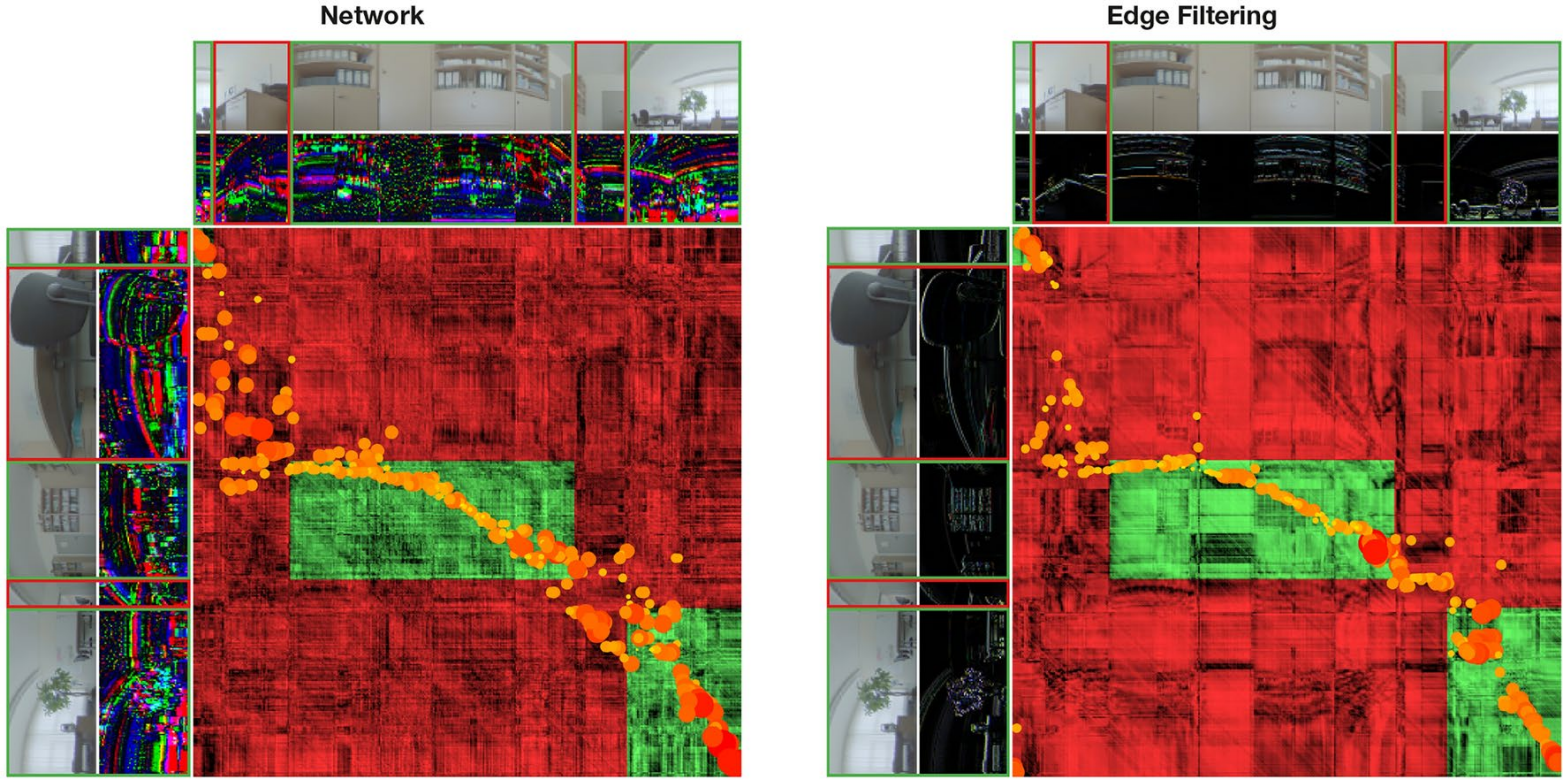
Searching columns with minimal distance in the scale plane stack in a situation with occlusion, but well textured image regions. The image pair is part of the Uni Office setting. The visualization follows the description for Figure 17. The angular error for MinWarping NP is 4.13°, and for MinWarping EF 0.34°.

To show that occlusion alone is not an issue for MinWarping EF, we look at Uni Office in Figure 18, a setting with low AAE and high RR for both MinWarping EF and NP. In this situation, large parts of matchable columns are occluded, but the remaining image regions offer distinctive details that compensate for mismatches and both methods find a solution with low error.

### Comparison of execution times

We compare the execution time of the proposed network-based image preprocessing with edge filtering and feature extraction using SuperPoint. For a comparison of execution times of MinWarping with two- and five-point algorithms for relative pose estimation, see the work of Fleer and Möller^53^.

Measurements are taken on a desktop computer with an Intel Core i9-10980XE CPU and an NVIDIA RTX 3090 GPU with Linux Kernel 6.9.7 and Python 3.11.4. We test both the execution times on the CPU and the GPU. Network-based image preprocessing and edge filtering are implemented using Tensorflow 2.12.0 and CUDA 11.8. SuperPoint uses Torch 2.4.1 and CUDA 12.1. The execution time is measured using the *default_timer* of the Python *timeit* module. The edge filtering and network-based image preprocessing are run with JIT compilation of Tensorflow enabled. Edge filtering is implemented in Tensorflow using the *roll* function, subtraction, and cutting off the topmost row of the image.

For every method, we take an identical sequence of 10001 randomly selected individual images (with a batch size of 1) from the dataset and measure the image processing time of a single method, including the copying time from host to device memory, if applicable. Because the first iteration of execution in Tensorflow includes the time for the JIT compilation, it is excluded from the time measurements. The results are shown in Table 2.

**Table 2:** Comparison of execution times of the presented methods. All values are in milliseconds.

Device	Method	Average time (ms)	Standard dev. (ms)	Max. time (ms)
CPU	Edge filtering	1.01	0.19	1.72
CPU	Preprocessing network (ours)	37.88	4.51	44.35
CPU	SuperPoint	207.17	10.18	283.90
GPU	Edge filtering	1.29	0.21	2.18
GPU	Preprocessing network (ours)	2.69	0.30	4.05
GPU	SuperPoint	11.55	3.84	22.61

## Discussion

We first discuss the network solution for environments with occlusion and few details, homing for short distances, and the results of the direct approach in comparison to the indirect relative pose estimation (RPE) pipeline. Finally, we review the achieved homing quality with the hybrid network architecture and the computational overhead of the proposed method.

### Occlusion and poorly textured environments

Occlusion does not pose a general problem for MinWarping EF, but the combination of occlusion and little details in the environment is difficult. MinWarping NP is able to improve here by adding noise to columns with few details, allowing it to focus on columns with meaningful information. We conclude that filtering landmarks is a key instrument in improving homing quality in these cases.

### Homing results for short distances

A notable percentage of failed runs in computation of the RR are due to loops created by home vectors close to the snapshot. Additionally, the AAE by distance for adjacent capture positions (0.2 m) shown in “Average angular error (AAE) by distance” is noticeably larger than the AAE for the whole dataset. This occasionally causes the robot to stop before it reaches the snapshot. Besides further improving homing quality for short distances, a possible solution may include avoiding recalculation of homing angle close to snapshot and instead using angles in the vicinity of the optimum of $\sim$ 1.6 m (relative to the room size). This could be realized using an image distance measure as an estimate of the physical distance between images.

### Homing quality of indirect relative pose estimation (RPE) pipeline

The AAE for the indirect RPE pipeline is similar to MinWarping EF, but the RR is close to MinWarping NP, meaning this method will reach the snapshot position with a similar reliability, but homing paths might not be as straight. For both Lab settings, Home Office, and Small Living Room, the indirect RPE pipeline even offers the highest RR out of the tested methods. These are environments that offer large amounts of salient features that are well distributed throughout the panoramic image. This is not the case for the challenging hallway settings, as large portions of the panoramic image show walls with few details. The problems in poorly textured environments are in line with the literature^1^. Furthermore, the homing quality for the Long Hallway setting also suffers from the room geometry described in “Average angular error (AAE) by setting”.

### Hybrid network architecture

Finally, we revisit the idea that learning a lean preprocessing network for homing has two main benefits, starting with homing quality improvements.

In “Average angular error (AAE) by setting and Inverse return ratio (IRR)”, we show that MinWarping with network-based preprocessing offers the lowest AAE and IRR when averaged over settings. The gap in homing quality between constant and mixed illumination is also reduced for both metrics. The homing quality improvements generalize to the unseen setting Computer Lab. Inspecting the highest 1% of angular errors, we observe an increase in high homing errors for one side of Short Hallway, and hypothesize that the uneven distribution of visible objects in this setting may be the issue. However, both the average angular error and the distribution of the highest angular errors provide an improvement over MinWarping with edge filtering.

We therefore conclude that the proposed hybrid architecture is a good fit for RPE in indoor scenes similar to the investigated home and office environments and can provide better reliability and straighter homing paths than the compared methods.

Regarding interpretability, we use a visual representation of the network output in conjunction with the homing process to show a primitive landmark filtering mechanism introduced by the network in “Analysis of network output”. However, the precise network mechanisms that lead to an improvement in landmark matching quality (considering illumination invariance) are yet unclear.

### Computational overhead

Following Table 2, the proposed network-based preprocessing is less demanding than feature extraction with SuperPoint, but when only a CPU is available, the computational overhead in comparison to edge filtering is large (on average 37.88 ms vs. 1.01 ms). However, the worst-case processing speed is 22 images/second, which can be sufficient for real-time image filtering.

Using the GPU reduces the execution time to an average of 2.69 ms and yields >246 images/second in the worst case, so using purpose-made hardware for network acceleration is recommended.

## Conclusion

In conclusion, combining the direct RPE method MinWarping with a neural network for image preprocessing yields a hybrid neural network architecture that improves upon the homing quality of MinWarping with edge filtering in home and office scenes. It also compares favorably with a modern deep-learning based indirect RPE pipeline in the same settings.

Fulfilling a secondary goal, we found HDR color images to be the optimal input for MinWarping with edge filtering.

The network output shows an unexpected interpretation of the input images: Not only are salient image features amplified in pixel value and size, but there is also noise added to image regions with few details. Analysing the network output in combination with the landmark matching process of MinWarping, we attribute the noisy image regions to a filtering mechanism that reduces the influence of image columns with few details.

The proposed hybrid architecture did not only allow for this kind of analysis, but also enabled us to limit the use of deep-learning to image processing. This reduces the number of network weights and computational overhead in comparison to a fully deep-learning-based approach, allowing for generalization to unseen data despite using a dataset of only moderate size.

## Supplementary Information

Supplementary Information.

## Data Availability

The image database, the pretrained image preprocessing model used in this work, and the corresponding source code will be made available at https://gitlab.ub.uni-bielefeld.de/tfwarp.
