# Supplementary material for: Relative pose estimation from panoramic images using a hybrid neural network architecture
Source: Sci Rep. 2024 Oct 24;14:25246. doi: 10.1038/s41598-024-75124-7 (PMC11502857; doi:10.1038/s41598-024-75124-7)
Supplement: Supplementary file 1 — Supplementary Information. [file 41598_2024_75124_MOESM1_ESM.pdf]

Relative Pose Estimation from Panoramic Images using a Hybrid Neural Network Architecture – Supplementary Information

Lars Offermann[1,*]

[1]Bielefeld University, Faculty of Technology, Bielefeld, 33615, Germany
[*]loffermann@techfak.uni-bielefeld.de

A Supplementary Information

S1 Siamese Architecture

We evaluate the network of Melekhov et al.[1] using a re-implementation in Tensorflow[2] and two toy examples to demonstrate the networks inability to predict the 2D homing angles.

For implementation, we chose the best-performing architecture from Melekhov et al.[1], but replace the two regression layers for translation (3D vector) and rotation (4D quaternion) with two regression layers for homing angles α and ψ. For this, we encode angles as vectors $(\cos(\alpha), \sin(\alpha))$ and retain the sum of squared errors as loss function.

Before training, we load the weights and biases for the CNN layers of the Hybrid-CNN[3] architecture (AlexNet[4] trained on a combination of the Places[3] and ImageNet[5] datasets) and freeze all trainable parameters such that only the regression part is affected during gradient descent.

As a toy example, we load image pairs $(\boldsymbol{S}, \boldsymbol{C})$ from the training dataset, but only use $\boldsymbol{S}$. We artificially create a new $\boldsymbol{C}$ by rotating $\boldsymbol{S}$ along the horizontal dimension, creating a camera rotation ψ at the same position without illumination changes. We rotate by integer steps, the amount is randomly chosen for each image in a batch. We only train the regression network for ψ. During training, we assess the SSE and the average angular error (AAE) for ψ within the batch. For the toy example, the SSE decreases fast, but the AAE for ψ oscillates around $\pi/2$, indicating that the network architecture is unable to predict the camera orientation beyond chance.

We then further reduce the complexity of the task by introducing an artificial landmark to $\boldsymbol{S}$ before rotation by replacing an image column with a the maximum value value of 1.0. When training with this modification, the artificial landmark is detected by the network and the AAE for ψ rapidly decreases.

We conclude that the network architecture is suitable for predicting the orientation of artificial patterns, but this ability does not translate to the natural images used in this work.

S2 Padding

There is no obvious optimal choice of padding the top and bottom edges when applying CNN kernels to the cylindrical panoramic image used in this work. We therefore test the padding types *reflect*, *symmetric*, *same*, *repeated*, and *valid* on the validation dataset. All other parameters are retained as described in Section 5.1. The type *symmetric* pads the tensor using a reflection of values through the top or bottom edge. *Reflect padding* also mirrors values, but through the top or bottom row of the input tensor. *Zero padding* fills the newly created border with zeros, while *repeat padding* repeats the input image in a cyclic manner.

All tests are run twice with different seeds to prevent overfit to the selection of validation data and random initialization of network weights. We assess the AAE over all validation data points, rounded to 2 decimal places. Based on the results shown in Table S1, we select the padding type *reflect* for all CNN layers. All further experiments are carried out with the network weights of seed 12.

Supp. Table S1. AAE on validation data for varying padding types and seeds.

Padding Type	AAE (rad) for Seed 6	AAE (rad) for Seed 12
Reflect	3.97	4.01
Symmetric	4.06	4.16
Same	4.23	4.4

Padding Type	AAE (rad) for Seed 6	AAE (rad) for Seed 12
Repeat	4.3	4.98
Valid	6.8	6.92

S3 Cutoff

A suitable cutoff was determined using a coarse-to-fine approach, first searching in 10° steps from 0° to 50°, then investigating the neighborhood of local minima 20° and 30° in 5° steps. The AAE was determined using the method described in Section 7.1, choosing HDR color images as input. For mixed illumination, we find a flat minimum for the AAE averaged over all settings between 15° to 35° with a total difference of 0.05°. Investigating constant illumination conditions between snapshot and current view, the minimum is located between 25° and 40°, also with a total difference of 0.05°. To save computation time, we choose 35° as the largest cutoff supported by both minima. This is a tradeoff in favor of average quality; the optimal cutoff choices per setting are often close to the extreme values of the investigated range, 0° and 50°.

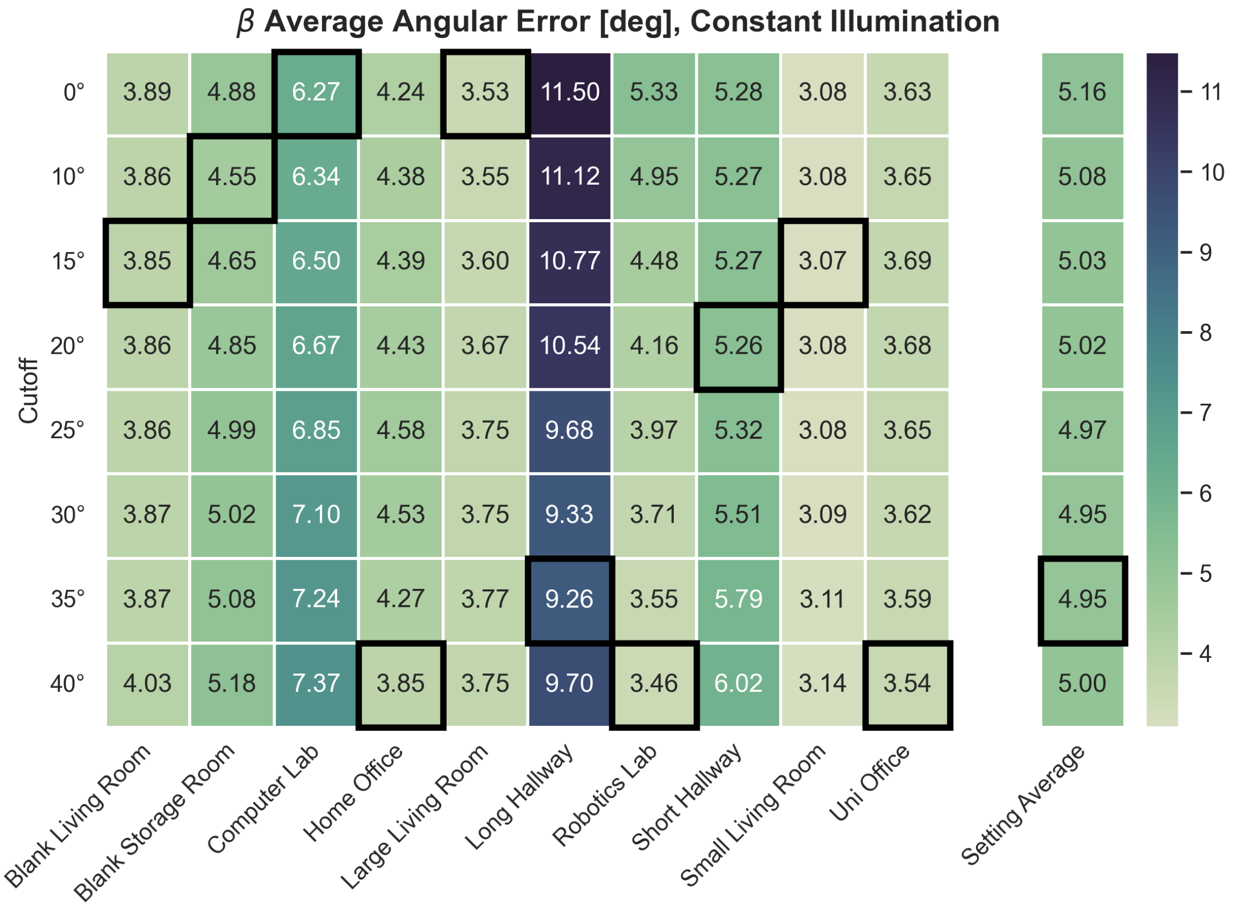

Supp. Figure S1. AAE for MinWarping with different cutoffs. Edge filtered HDR color images are used as input images. Illumination between snapshot and current view is constant. All values are in degrees. The best results for each setting are marked with a black border.

References

1. Melekhov, I., Ylioinas, J., Kannala, J. & Rahtu, E. Relative camera pose estimation using convolutional neural networks. In Blanc-Talon, J., Penne, R., Philips, W., Popescu, D. C. & Scheunders, P. (eds.) *Advanced Concepts for Intelligent Vision Systems - 18th International Conference, ACIVS 2017, Antwerp, Belgium, September 18-21, 2017, Proceedings*, vol. 10617 of *Lecture Notes in Computer Science*, 675–687, DOI: 10.1007/978-3-319-70353-4_57 (Springer, 2017).

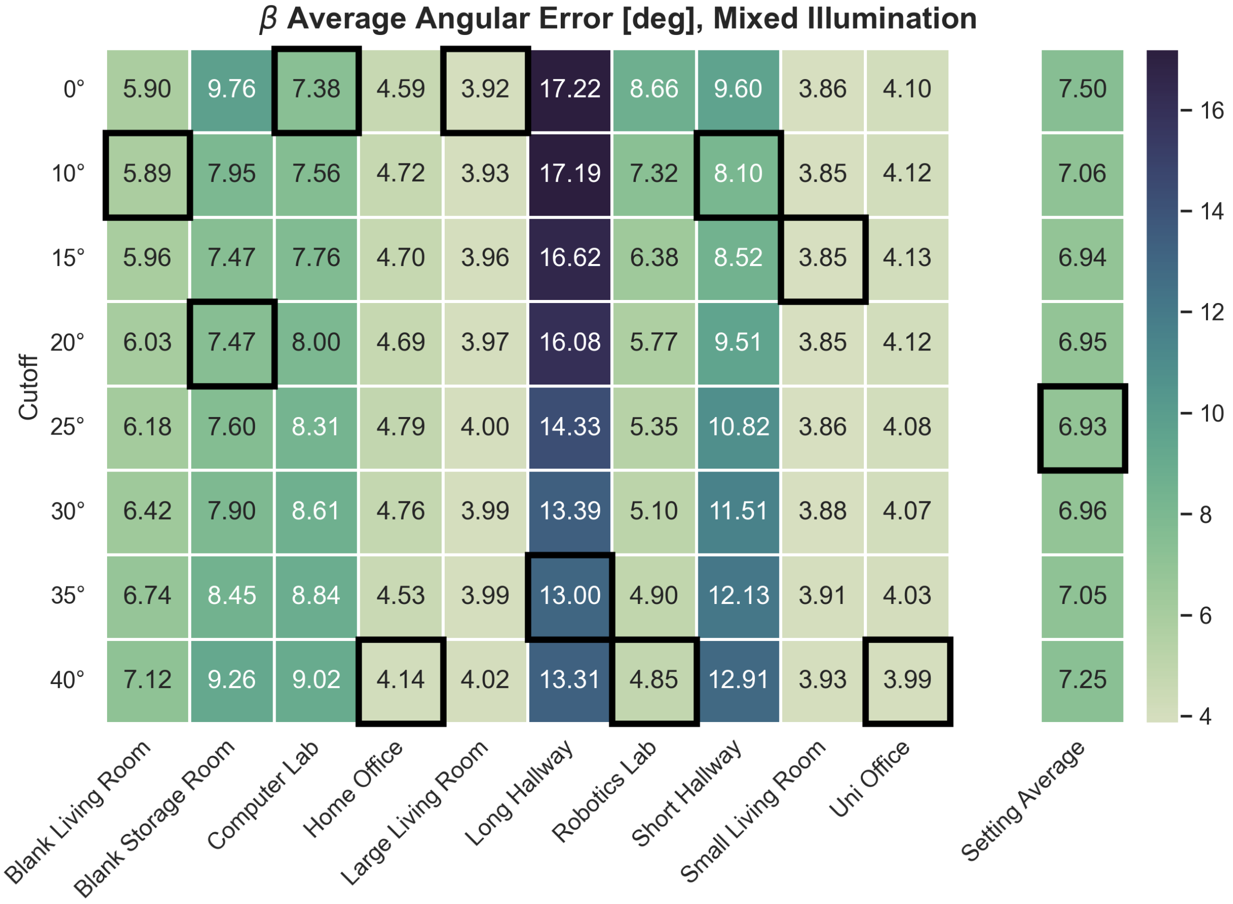

Supp. Figure S2. AAE for MinWarping with different cutoffs. Edge filtered HDR color images are used as input images. Illumination between snapshot and current view is mixed. All values are in degrees. The best results for each setting are marked with a black border.

2. Abadi, M. *et al.* TensorFlow: Large-scale machine learning on heterogeneous systems (2015). Software available from tensorflow.org.

3. Zhou, B., Lapedriza, À., Xiao, J., Torralba, A. & Oliva, A. Learning deep features for scene recognition using places database. In Ghahramani, Z., Welling, M., Cortes, C., Lawrence, N. D. & Weinberger, K. Q. (eds.) *Advances in Neural Information Processing Systems 27: Annual Conference on Neural Information Processing Systems 2014, December 8-13 2014, Montreal, Quebec, Canada*, 487–495 (2014).

4. Krizhevsky, A., Sutskever, I. & Hinton, G. E. Imagenet classification with deep convolutional neural networks. *Commun. ACM* **60**, 84–90, DOI: 10.1145/3065386 (2017).

5. Deng, J. *et al.* Imagenet: A large-scale hierarchical image database. In *2009 IEEE Computer Society Conference on Computer Vision and Pattern Recognition (CVPR 2009), 20-25 June 2009, Miami, Florida, USA*, 248–255, DOI: 10.1109/CVPR.2009.5206848 (IEEE Computer Society, 2009).
